# ﻿Peruvian nudibranchs (Mollusca, Gastropoda, Heterobranchia): an updated literature review-based list of species

**DOI:** 10.3897/zookeys.1176.103167

**Published:** 2023-08-23

**Authors:** Alessandra Grández, André Ampuero, Sergio P. Barahona

**Affiliations:** 1 Carrera de Biología Marina, Universidad Científica del Sur, Lima, Perú Universidad Científica del Sur Lima Peru

**Keywords:** biogeography, bibliographic compilation, geographic distribution, Nudibranchia, Peru, sea slug, taxonomy

## Abstract

Nudibranchs, as a group, have received limited attention in terms of scientific study along the coastline of Peru. Here, an updated and comprehensive list of nudibranch species found in the Peruvian sea is presented, compiled through an extensive review of relevant literature. This compilation encompasses a total of 31 species, classified into two suborders, 10 superfamilies, 20 families, and 28 genera. With respect to the biogeographic provinces along the Peruvian coast, 23 species inhabit the Warm Temperate Southeastern Pacific province, 18 species occur in the Tropical Eastern Pacific province, and 10 species are found in both provinces, crossing the transitional zone between them. In terms of distribution patterns, two species exhibit a cosmopolitan distribution (*Glaucusatlanticus* and *Fionapinnata*), while two species display a circumtropical distribution (*Cephalopygetrematoides* and *Phylliroebucephala*). One species exhibits a bipolar distribution in the Eastern Pacific and possesses an amphi-South American distribution (*Rostangapulchra*). Additionally, six species exhibit an amphi-South American distribution (*Rostangapulchra*, *Diaululapunctuolata*, *Dotouva*, *Tyrinnaevelinae*, *Tyrinnadelicata*, and *Dorisfontainii*), and two species are endemic to Peru (*Corambemancorensis* and *Felimaresechurana*). This study provides comprehensive information on biogeographical aspects, geographical distributions, and taxonomic updates within the nudibranch species documented in Peru. Furthermore, we discuss the status of species listed in previous literature that have not been confirmed by collections, referring to them as potentially occurring species.

## ﻿Introduction

Nudibranchia Cuvier, 1817 (Subclass Heterobranchia, Infraclass Euthyneura, Superorder Nudipleura) represents an order of exclusively marine gastropod mollusks, distinguished by the absence of shells in the adult stage (Behrens et al. 2005; Wägele and Klussmann-Kolb 2005). These remarkable organisms exhibit striking aposematic body colorations, making them frequent targets of underwater photography. Approximately 3000 species of nudibranchs have been described worldwide inhabiting both cold and tropical regions (Shields 2009; Almada et al. 2016), primarily in shallow waters ranging from 0 to 30 m in depth (Wägele and Klussmann-Kolb 2005). However, novel species discoveries have expanded our knowledge of nudibranchs in deeper habitats (Valdés 2001a, 2001b; Gosliner et al. 2008). The ecological importance of nudibranchs stems from their role in controlling populations of cnidarians and poriferans, from which they acquire and incorporate toxins for their own defense (Greenwood 2009; Faulkner and Ghiselin 1983). Furthermore, recent research has elucidated symbiotic associations between nudibranchs and bacteria, wherein the bacteria provide essential nutrients to the hosts (Zhukova et al. 2022). Certain species have been found to synthesize secondary metabolites with high biotechnological potential (Pereira et al. 2012; Dean and Prinsep 2017). Additionally, nudibranch species possess the potential to serve as environmental indicators, as they are sensitive to ocean stressors such as coastal pollution (Caballer et al. 2008).

The most recent inventory of aquatic mollusks in Peru, as documented by Ramírez et al. (2003), recorded a total of 1018 marine species, primarily comprising gastropods and bivalves, with only a limited number of nudibranch species reported. In fact, the Peruvian sea is recognized as one of the impoverished regions worldwide in terms of nudibranch diversity (Schrödl 1997, 2002, 2003; Schrödl and Hooker 2014). In comparison, other regions such as the Caribbean Sea, the Tropical Eastern Pacific, the Indian Ocean, the Mediterranean Sea (Sachidhanandam et al. 2000; Valdés et al. 2006; Chavanich et al. 2013; Ah Shee Tee et al. 2019; Furfaro et al. 2020; Londoño-Cruz 2021), as well as other South American countries including Brazil, Chile, and Venezuela (Fischer and Cervera 2005b; Ardila et al. 2007; Aldea et al. 2011; Padula et al. 2011; Alvim and Pimenta 2013; Gutiérrez et al. 2015; Araya and Valdés 2016; Londoño-Cruz 2021) exhibit considerably higher richness of nudibranch species.

The earliest records of nudibranchs in Peru can be attributed to d’Orbigny (1835–1846) and later to Dall (1909). Following a significant period without the discovery of new species, Millen et al. (1994) reported the presence of *Okenialuna* in Peruvian and Chilean waters. Subsequently, the first list of Peruvian aquatic mollusks was published, which included some nudibranch species (Álamo and Valdivieso 1997), and two years later, another list was published (Paredes et al. 1999), in which Sandra Millen was acknowledged for the preliminary list of species belonging to the infraclass Opisthobranchia (a taxonomic category that has since been abandoned and deprecated; see Jörger et al. 2010; Schrödl et al. 2011; Wägele et al. 2014). In 2003, an updated inventory of Peruvian aquatic mollusks was published (Ramírez et al. 2003), which included the nudibranch species reported up to that time. Several years later, four new species were reported on the northern coast of Peru, *Felimidabaumanni*, *Doriopsillajanaina*, *Kynariacynara*, and *Cuthona* sp. (Nakamura 2006), with the species *Corambemancorensis* identified as endemic (Martynov et al. 2011), and the species *Spurillaneapolitana* (later corrected as *Spurillabraziliana*) (Uribe and Pacheco 2012). Subsequently, four additional nudibranch species were documented for the Peruvian coast (Uribe et al. 2013), and another study focused on the species shared with Chile (Schrödl and Hooker 2014). *Felimaresechurana* was identified as an endemic species in the transition zone of the northern coast (Hoover et al. 2017) and, additionally, two new species of planktonic nudibranchs were described (Quesquen 2017).

We must emphasize that certain species have been listed in previous publications as occurring in Peru without sufficient evidence, such as assumptions of geographic continuity (e.g., *Cadlinasparsa*; Álamo and Valdivieso 1997), reliance on personal communications only (e.g., Polyceracf.alabe; Paredes et al. 1999; Uribe et al. 2013), and misinterpretations (e.g., *Gargamellaimmaculata* and *Thecaceradarwini*; Nakamura 2006), which has created problems as these listings have persisted in the literature. To distinguish them from the confirmed species, the term “potentially occurring” is used hereafter. However, such statuses can be revised in the future, as exemplified by the species *Rostangapulchra*, which was initially predicted for Peruvian waters for many years until its confirmation (Schrödl and Hooker 2014).

El Niño-Southern Oscillation (ENSO) warm events have been observed to induce southward displacement of tropical species (Velez and Zeballos 1985; Paredes et al. 1998) while cold events tend to enhance the intensity of the Humboldt Current, resulting in the northward transport of larvae. Specifically, the northward transport of larvae by the Humboldt Current or the southward transport facilitated by warm ENSO events may introduce Magellanic or tropical species, respectively, into Peruvian waters, thereby influencing distribution ranges. In addition, the susceptibility of nudibranchs to temperature fluctuations, particularly during their larval stages (Leatherman 2019) due to the aragonite-based internal structure found in several species (Ehrlich 2010), their small body size, limited populations (Nybakken 1978), and the sensitivity of the Humboldt Current Ecosystem to oceanic stressors (Echevin et al. 2012) such as warming and acidification (Barnosky et al. 2011; Ceballos et al. 2015; Pievani 2014), collectively suggest that the diversity and distribution of nudibranchs could be impacted (Nimbs and Smith 2018).

The available information on Peruvian nudibranchs remains limited primarily due to a lack of research effort (Uribe and Pacheco 2012). Explorations specifically targeting nudibranchs have been extremely scarce, and most sightings and reports are sporadic (Nakamura 2006; Schrödl and Hooker 2014; Uribe et al. 2013). Given that the species richness of nudibranchs is likely underestimated in Peru (Hooker pers. comm.), this taxonomic order warrants further attention. Our aim was to update and revise the list of nudibranchs in the Peruvian sea, based on a comprehensive review of the scientific literature.

## ﻿Materials and methods

A comprehensive review was conducted to compile all available literature pertaining to the order Nudibranchia in Peru. The literature search encompassed diverse sources of information, including peer-reviewed journal articles, books, book chapters, “grey literature” (such as scientific reports and theses), and the Sea Slug Forum (http://www.seaslugforum.net/). Key terms such as ‘Opisthobranchia,’ ‘Heterobranchia,’ ‘Nudibranch,’ ‘Nudibranchia,’ ‘sea slug,’ ‘phylogeny,’ ‘checklist,’ ‘Peru,’ ‘Humboldt,’ and ‘taxonomy’ were employed. Pertinent data, such as type material, geographic distribution, sampling/reporting sites, bathymetric distribution, and biogeographical provinces, were meticulously included. The most up-to-date scientific names were validated through the World Register of Marine Species (WoRMS, https://www.marinespecies.org/), and reports (occurrences) were cross-referenced using the Global Biodiversity Information Facility (GBIF, https://www.gbif.org/) and the iNaturalist database (https://www.inaturalist.org/). Any modifications, revalidations, or refutations pertaining to taxonomy are concisely presented as “Remarks”, accompanied by justifications as needed. Endemic species of Peru are also duly indicated. The distribution map was made using QGIS 3.22.8 software (QGIS Development Team 2022), while VENNY 2.1 online software (Oliveros 2016) was employed to visualize the number of species shared with some neighboring countries. Potentially occurring species were clearly distinguished from the confirmed ones.

The acronyms corresponding to the collections where the type material for certain species is deposited have been included, as follows:

**CASIZ**California Academy of Sciences Invertebrate Zoology, San Francisco

**CZA** Colección de Zoología Acuática, Universidad Peruana Cayetano Heredia, Lima

**NHMUK**Natural History Museum, London

**MHNURP** Museo Historia Natural Vera Alleman Haeghebaert, Universidad Ricardo Palma, Santiago de Surco

**RMNH**Naturalis Biodiversity Center, Leiden

**SMNH**Swedish Museum of Natural History, Stockholm

**USNM**Smithsonian National Museum of Natural History, Washington DC

**ZMB**The Berlin Zoological Museum, Berlin

**ZSM**The Bavarian State Collection of Zoology, Munich

## ﻿Results

### ﻿Overview

**Figure 1.**
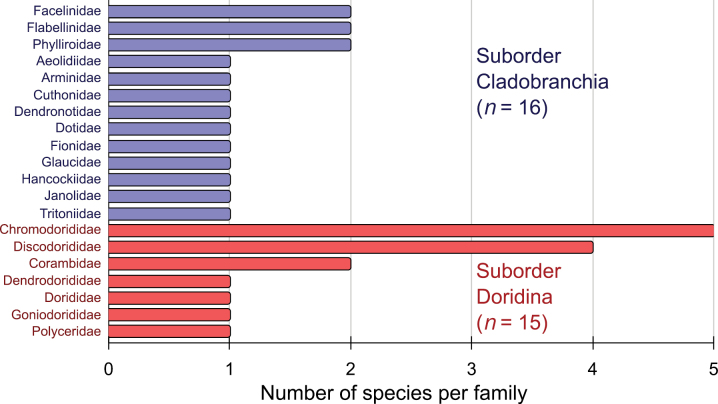
Number of species by family and suborder of Nudibranchia in Peruvian waters.

**Figure 2.**
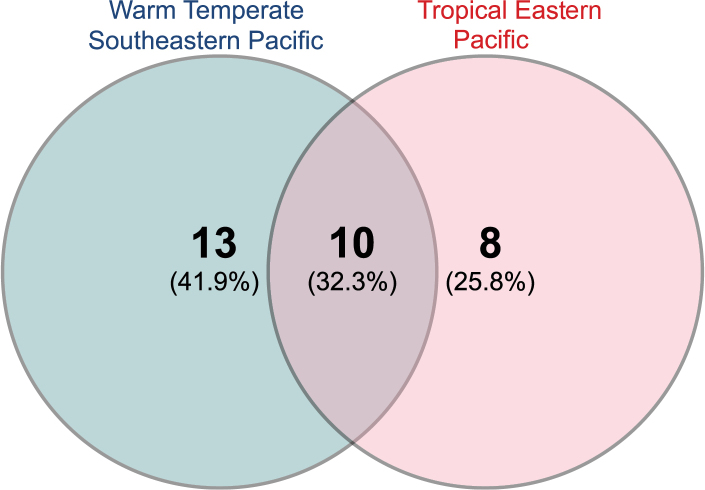
Venn diagram showing the number of species shared between the two coastal marine biogeographic provinces present in Peru.

A total of 31 species, encompassing two suborders, ten superfamilies, 20 families, and 28 genera (Table 1), has been confirmed within Peruvian waters. The suborder Cladobranchia comprises 16 species, spanning five superfamilies and 13 families. The suborder Doridina consists of 15 species, distributed among five superfamilies and seven families (Table 1). Notably, the families Chromodorididae and Discodorididae, both belonging to the suborder Doridina, exhibit remarkable species richness with five and four species, respectively (Fig. 1). Additionally, potentially occurring species (*n* = 9) are distributed across five superfamilies, eight families, and nine genera (Table 2). A chronological overview of articles documenting nudibranch species in the Peruvian sea can be found in Table 3, revealing the progressive increase in reported species and the latest updates in scientific nomenclature. Regarding the distribution of species along the Peruvian coast, according to the coastal marine biogeographical classification proposed by Spalding et al. (2007), 23 species inhabit within the Warm Temperate Southeastern Pacific province, 18 species are found within the Tropical Eastern Pacific province, and ten species are common to both provinces (Table 4, Fig. 2).

**Table 1.** Nudibranch species confirmed for Peruvian waters according to the bibliographic compilation of this study.

Suborders (*n* = 2)	Superfamilies (*n* = 10)	Families (*n* = 20)	Species (*n* = 31)
Cladobranchia	Aeolidioidea	Aeolidiidae	*Spurillabraziliana* MacFarland, 1909
		Facelinidae	*Phidianalottini* (Lesson, 1831)
			*Bajaeolisbertschi* Gosliner & Behrens, 1986
		Glaucidae	*Glaucusatlanticus* Forster, 1777
	Arminoidea	Arminidae	*Arminacalifornica* (J.G. Cooper, 1863)
	Dendronotoidea	Dendronotidae	Dendronotuscf.venustus MacFarland, 1966
		Dotidae	*Dotouva* Er. Marcus, 1955
		Hancockiidae	*Hancockiaschoeferti* Schrödl, 1999
		Phylliroidae	*Cephalopygetrematoides* (Chun, 1889)
			*Phylliroebucephala* Lamarck, 1816
		Cuthonidae	*Cuthona* sp.
		Fionidae	*Fionapinnata* (Eschscholtz, 1831)
		Flabellinidae	*Kynariacynara* (Ev. Marcus & Er. Marcus, 1967)
			*Coryphellinacerverai* (M. A. Fischer, van der Velde & Roubos, 2007)
	Proctonotoidea	Janolidae	*Janolusrebeccae* Schrödl, 1996
	Tritonioidea	Tritoniidae	*Tritonia* sp.
Doridina	Chromodoridoidea	Chromodorididae	*Tyrinnadelicata* (Abraham, 1877)
			*Tyrinnaevelinae* (Er. Marcus, 1958)
			*Felimareagassizii* (Bergh, 1894)
			*Felimaresechurana* Hoover, Padula, Schrödl, Hooker & Valdés, 2017
			*Felimidabaumanni* (Bertsch, 1970)
	Doridoidea	Discodorididae	*Baptodorisperuviana* (d’Orbigny, 1837)
			*Diaululavariolata* (d’Orbigny, 1837)
			*Diaululapunctuolata* (d’Orbigny, 1837)
			*Rostangapulchra* MacFarland, 1905
		Dorididae	*Dorisfontainii* d’Orbigny, 1837
	Onchidoridoidea	Corambidae	*Corambelucea* Er. Marcus, 1959
			*Corambemancorensis* Martynov et al., 2011
		Goniodorididae	*Okenialuna* Millen, Schrödl, Vargas & Indacochea, 1994
	Phyllidioidea	Dendrodorididae	*Doriopsillajanaina* Er. Marcus & Ev. Marcus, 1967
	Polyceroidea	Polyceridae	*Polycerapriva* Er. Marcus, 1959

**Table 2.** Nudibranch species that could potentially occur in Peruvian waters based on the bibliographic compilation of this study.

Suborder (*n* = 2)	Superfamilies (*n* = 5)	Families (*n* = 8)	Species (*n* = 9)
Cladobranchia	Aeolidioidea	Aeolidiidae	*Aeolidiacampbellii* (Cunningham, 1871)
		Glaucidae	*Glaucus* sp.
		Phylliroidae	*Phylliroelichtensteinii* Eschscholtz, 1825
	Fionoidea	Coryphellidae	*Itaxiafalklandica* (Eliot, 1907)
	Flabellinoidea	Flabellinidae	*Coryphellinamarcusorum* (Gosliner & Kuzirian, 1990)
Doridina	Chromodoridoidea	Cadlinidae	*Cadlinasparsa* (Odhner, 1922)
		Discodorididae	*Gargamellaimmaculata* Bergh, 1894
	Polyceroidea	Polyceridae	Polyceracf.alabe Collier & Farmer, 1964
			*Thecaceradarwini* Pruvot-Fol, 1950

**Table 3.** Chronologically ordered publications listing nudibranch species in the Peruvian sea. Legend: First reports for Peruvian waters: ^a^d'Orbigny (1835–1846), ^b^Dall (1909), ^c^Millen et al. (1994), ^d^Nakamura (2006), ^e^Schrödl (2003), ^f^Schrödl (1999), ^g^Schrödl (2000), ^h^Martynov et al. (2011), ^i^Uribe et al. (2013), ^j^Uribe and Pacheco (2012), ^k^Schrödl and Hooker (2014), ^l^Hoover et al. (2017), ^m^Quesquen (2017), sp = The total count of nudibranch species mentioned in each checklist. Single asterisk (*) shows potentially occurring species and double asterisk (**) shows recent confirmation of previously predicted species in Peruvian waters. ^§^ shows that the scientific name has undergone changes.

d’Orbigny (1835–1846) (sp = 5)	Dall (1909) (sp = 6)	Álamo and Valdivieso (1997)	Paredes et al. (1999) / Ramírez et al. (2003) (sp = 18)	(Nakamura 2006) (sp = 23)	Uribe et al. (2013) (sp = 25)	Schrödl and Hooker (2014) (sp = 14)	This study (sp = 31)
							* nine potentially ocurring species
		(sp = 7)					
*Doriopsisperuviana* ^a^	*Dorisperuviana*	*Dendrodorisperuviana*	*Dorisperuviana*	*Baptodorisperuviana*	*Baptodorisperuviana*	*Baptodoris? peruviana*	*Baptodorisperuviana* ^§^
*Diphyllidiacuvieri*	*Pleurophyllidiacuvieri*			*Arminacuvieri*	*Arminacalifornica*		*Arminacalifornica* ^§^
*Phidiananatans* ^a^	*P.natans*/*Fionapinnata*	*Phidiananatans*	*P.natans* / *Fionapinnata*	*Fionapinnata*	*Fionapinnata*		*Fionapinnata* ^§^
*Phidianainca* ^a^	*Phidianainca*	*Phidianainca*	*Phidianalottini*	*Phidianalottini*	*Phidianalottini*	*Phidianalottini*	*Phidianalottini* ^§^
*Glaucusdistichoicus*	*Glaucusdistichoicus*		*Glaucusatlanticus*		*Glaucusatlanticus*		*Glaucusatlanticus* ^§^
	*Dorispunctuolata* ^b^	*Dorispunctuolata*	*Anisodorispunctuolata*	*Diaululapunctuolata*			*Diaululapunctuolata* ^§^
		*Okenialuna* ^c^	*Okenialuna*	*Okenialuna*	*Okenialuna*	*Okenialuna*	*Okenialuna*
		Cadlina? sparsa*	Cadlinasparsa*	Cadlinasparsa*			Cadlinasparsa*
		Rostangapulchra*	Rostangapulchra*	Rostangapulchra*		Rostangapulchra**	*Rostangapulchra*
			Aeolidiaserotina*	Aeolidiaserotina*			Aeolidiacampbellii*^§^
			Hypselodoriscf.agassizii	*Hypselodorisagassizii*	*Felimareagassizii*		*Felimareagassizii* ^§^
			Flabellinacf.falklandica*	*Flabellinafalklandica*			Itaxiafalklandica*^§^
			*Dendronotusfrondosus*	*Dendronotusfrondosus*	Dendronotuscf.venustus		Dendronotuscf.venustus ^§^
			Dotocf.uva	*Dotouva*	*Dotouva*	*Dotouva*	*Dotouva*
			Polyceracf.alabe	*Polyceraalabe*	*Polyceraalabe*		Polyceracf.alabe
			*Tyrinnaevelinae*	*Tyrinnaevelinae*	*Tyrinnaevelinae*		*Tyrinnaevelinae*
			*Bajaeolusbertschi*	*Bajaeolisbertschi*	*Bajaeolisbertschi*		*Bajaeolisbertschi*
			Phylliroelichtensteini*				Phylliroelichtensteinii*
				*Flabellinacynara* ^d^	*Flabellinacynara*		*Kynariacynara* ^§^
				*Glossodorisbaumanni* ^d^	*Glossodorisbaumanni*		*Felimidabaumanni* ^§^
				*Cuthona* sp.^d^	*Cuthona* sp.		*Cuthona* sp.
				*Doriopsillajanaina* ^d^	*Doriopsillajanaina*		*Doriopsillajanaina*
				*Flabellina* sp. 2^e^	*Flabellinacerverai*	Flabellinacf.cerverai	*Coryphellinacerverai* ^§^
				Gargamellaimmaculata*^f^			Gargamellaimmaculata*
				*Dorisfontainei* ^g^	*Dorisfontainei*	*Dorisfontainei*	*Dorisfontainii* ^§^
					*Corambemancorensis* ^h^		*Corambemancorensis*
					*Diaululavariolata* ^i^	*Diaululavariolata*	*Diaululavariolata*
					*Tyrinnanobilis* ^i^		*Tyrinnadelicata* ^§^
					*Tritonia* sp.^i^		*Tritonia* sp.
					Spurillacf.neapolitana ^j^	*Spurillabraziliana*	*Spurillabraziliana*
				Thecaceradarwini*	Thecaceradarwini*	Thecaceradarwini*	Thecaceradarwini*
						*Polycerapriva* ^k^	*Polycerapriva*
						*Corambelucea* ^k^	*Corambelucea*
						*Janolusrebeccae* ^k^	*Janolusrebeccae*
						*Hancockiaschoeferti* ^k^	*Hancockiaschoeferti*
							*Felimaresechurana* ^l^
							*Cephalopygetrematoides* ^m^
							*Glaucus* sp.*^m^
							*Phylliroebucephala* ^m^
							Coryphellinamarcusorum*

**Table 4.** Presence of nudibranch species inhabiting Peruvian waters along several marine coastal biogeographic provinces according the reporting sites. Legend: the asterisk (*) indicates potentially occurring species in Peruvian waters.

Families	Species	Magellanic	Pacific					Atlantic						Mediterranean Sea	Circumtropical	Cosmopolitan
			Warm Temperate Southeastern Pacific	Tropical Eastern Pacific	Warm Temperate Northeast Pacific	Cold Temperate Northeast Pacific	Galapagos	North Brazil Shelf	Tropical Northwestern Atlantic	Tropical Southwestern Atlantic	West African Transition	Warm Temperate Southwestern Atlantic	Lusitanian			
Aeolidiidae	*Spurillabraziliana*		X	X	X				X	X						
	*Aeolidiacampbellii* (*)	X	X													
Facelinidae	*Phidianalottini*	X	X													
	*Bajaeolisbertschi*			X	X											
Glaucidae	*Glaucusatlanticus*		X	X	X		X		X	X				X	X	X
	*Glaucus* sp. (*)		X													
Arminidae	*Arminacalifornica*			X	X	X										
Dendronotidae	Dendronotuscf.venustus		X		X	X										
Dotidae	*Dotouva*	X	X									X				
Hancockiidae	*Hancockiaschoeferti*	X	X													
Phylliroidae	*Cephalopygetrematoides*		X	X	X						X		X		X	
	*Phylliroebucephala*		X	X					X				X	X	X	
	*Phylliroelichtensteinii* (*)		X							X						X
Coryphellidae	*Itaxiafalklandica* (*)	X	X									X				
Cuthonidae	*Cuthona* sp.	X		X												
Fionidae	*Fionapinnata*		X													X
Flabellinidae	*Kynariacynara*		X	X	X											
	*Coryphellinacerverai*		X	X												
	Coryphellinamarcusorum (*)			X					X							
Janolidae	*Janolusrebeccae*		X	X												
Tritoniidae	*Tritonia* sp.		X	X												
Cadlinidae	*Cadlinasparsa* (*)	X	X		X											
Chromodorididae	*Tyrinnadelicata*	X	X													
	*Tyrinnaevelinae*			X	X											
	*Felimareagassizii*		X	X	X		X									
	*Felimaresechurana*			X												
	*Felimidabaumanni*			X	X											
Discodorididae	*Baptodorisperuviana*		X													
	*Diaululavariolata*	X	X													
	*Diaululapunctuolata*		X													
	*Rostangapulchra*	X	X		X	X										
	*Gargamellaimmaculata* (*)	X	X													
Dorididae	*Dorisfontainii*	X	X													
Corambidae	*Corambelucea*	X	X	X												
	*Corambemancorensis*			X												
Goniodorididae	*Okenialuna*		X													
Dendrodorididae	*Doriopsillajanaina*			X	X		X									
Polyceridae	*Polycerapriva*	X	X													
	Polyceracf.alabe (*)			X	X											
	*Thecaceradarwini* (*)	X	X													

**Figure 3.**
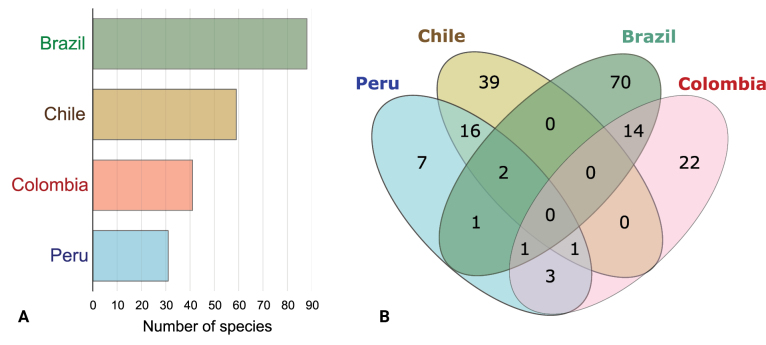
**A** Nudibranch species richness by country **B** Venn diagram illustrating the shared species count among countries. Only the confirmed species from Peru were considered. The counts of nudibranch species for neighboring countries were derived from a comprehensive literature review (data not shown).

**Figure 4.**
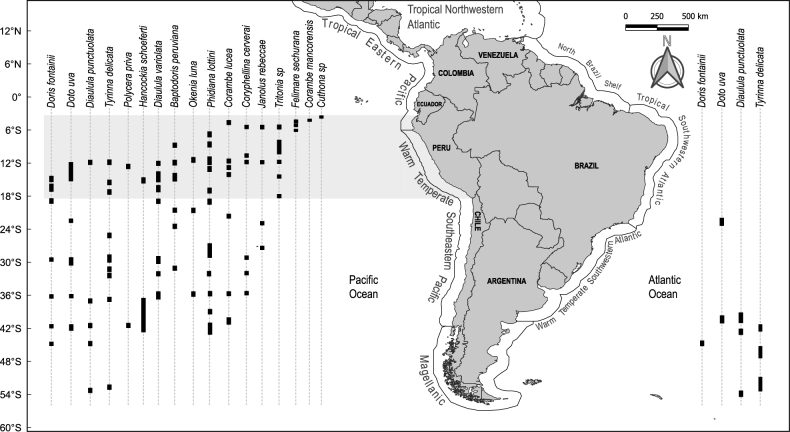
Reporting sites for nudibranch species found in Peruvian waters whose geographic ranges are limited to South America. The gray band highlights the locations where these species have been reported along the Peruvian coastline. The coastal marine biogeographic classification introduced by Spalding et al. (2007) is presented to provide context.

Peru’s inventory of nudibranch species is comparatively modest in comparison to other South American countries, such as Chile, Colombia, and Brazil (Fig. 3A). Concerning species shared with these countries, of the 31 species that have been conclusively documented in Peruvian waters, 19 species are found in Chilean waters, four species in Colombian waters, and four species in Brazilian waters (Fig. 3B). The recorded collection/reporting sites of nudibranch species found in Peruvian waters, limited to South America, within the framework of the coastal-marine biogeographical classification proposed by Spalding et al. (2007), are illustrated in Fig. 4.

### ﻿﻿Confirmed species in Peruvian waters

#### ﻿Suborder Cladobranchia

**Superfamily Aeolidioidea Gray, 1827**

**Family Aeolidiidae Gray, 1827**

##### ﻿ Spurilla braziliana

Taxon classificationAnimaliaNudibranchiaAeolidiidae

MacFarland, 1909

BE76A3A4-19B6-5DDB-8C13-FEDFFE3E787D

Peruvian specimen photographs: Uribe and Pacheco (2012) , Uribe et al. (2013) Common name: Brazilian Aeolid

###### Habitat.

Benthic.

###### Depth.

0–10 m (Gosliner 1979; Uribe and Pacheco 2012).

###### Type material.

Holotype CASIZ 019731–Alagoas, Brazil (Carmona et al. 2014).

###### Distribution.

This species exhibits a distribution range spanning the western Atlantic, extending from Florida to Brazil (Behrens and Hermosillo 2005; Marcus 1959), as well as the Pacific Ocean.

###### Sampling/reporting sites.

In Peru, it was reported in Ferrol Bay (Chimbote, 09°06'S) (Uribe and Pacheco 2012) and Pucusana (Lima, 12°25'S) (Uribe et al. 2013) under the name *Spurillaneapolitana*. In the western Pacific, this species has been reported in Japan (Hamatani 2000), China (Lin 1992), and Australia (Willan 2006). Within the Eastern Pacific, it was reported in Mexico, Costa Rica, and Colombia (Carmona et al. 2013). Additionally, a specimen was reported from Hawaii (Gosliner 1979).

###### Remarks.

Carmona et al. (2013), based on mitochondrial and nuclear sequences, revealed that *Spurillaneapolitana* MacFarland, 1909 comprises a complex of five cryptic species. In the light of this discovery, the name *Spurillabraziliana* has been assigned to populations found in the western Atlantic and Pacific regions. Carmona et al. (2013) further speculated that the occurrence of this species in the Pacific Ocean might be attributed to human-mediated introductions.

**Family Facelinidae Bergh, 1889**

##### ﻿ Phidiana lottini

Taxon classificationAnimaliaNudibranchiaAeolidiidae

(Lesson, 1831)

D63BDE3E-FD27-5B0A-99FB-5DC3C37055F1

**Figure 5.**
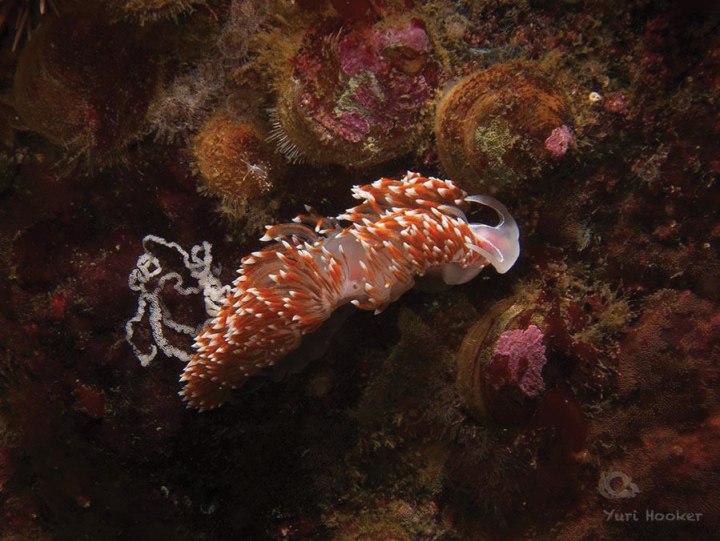
*Phidianalottini*, courtesy of Yuri Hooker.

Peruvian specimen photographs: Fig. 5

###### Habitat.

Benthic.

###### Depth.

0–15 m (Schrödl and Hooker 2014).

###### Type material.

Not available.

###### Distribution.

From Puerto Malabrigo (La Libertad, Peru, 07°42'S) (Flores 2014) to Melinka (Guaitecas islands, Chilean fjord region, 43°52'S) (Schrödl 2003).

###### Sampling/reporting sites.

In Peru, it was initially reported in Callao as *Phidianainca* by d’Orbigny, (1835–1846) and Dall (1909). It was also reported in Isla Santa (Ancash, 09°01'S), Ancon (Lima, 11°47'S), San Bartolo (12°22'S), Pucusana (Lima, 12°25'S) and San Juan de Marcona (Ica, 15°21′S) (Uribe et al. 2013). Schrödl and Hooker (2014) also collected individuals in Pucusana (12°25'S), Paracas (13°48'S) and La Punta (Callao). The report by Flores (2014) in Puerto Malabrigo (La Libertad, 07°42’S) revealed the northernmost locality of this species distribution. Valdivia-Chavez et al. (2020) presented a recent report of this species in Arequipa (15–17°S). Other reports (occurrences) for this species in the Peruvian coast are Playa Tartacay (El Paraíso, Huaura, Lima, 11°13'44.9"S) (Zanabria 2020e), Isla Chuncho (Pucusana, Lima, 12°28'S) (Guzman 2018a; Cuba 2019) and Puerto General San Martín (Paracas Bay, Pisco, Ica, 13°48'37.3"S) (Zanabria 2020d). In Chile, it was reported in Playa Brava (Caldera, Atacama, 27°03'S), Calderilla (Atacama, 27°05'S) (Araya and Valdés 2016), Punta Blanca (Arica, 18°29'S), Comau Fjord (42°15'S), and Melinka (Guaitecas islands, Chilean fjord region, 43°52'S) (Schrödl 2003). Other reports (occurrences) for this species in the Chilean coast include Chascos Bay (Copiapó, Atacama, 27°40'S), Reserva Nacional Pingüino de Humboldt (Huasco, Atacama, 29°01'S), Coquimbo (29°58'S), Valparaíso (33°21'S), Concepción (36°45'S), Valdivia (39°57'S), and Chiloé (41°53'S) (iNaturalist 2023a).

###### Remarks.

Initially designated as *Phidianainca* (d’Orbigny, 1837) until research by Schrödl (1997) who considered it conspecific with *Eolidialottini* Lesson, 1831, proposing *Phidianalottini* as a valid name.

##### ﻿ Bajaeolis bertschi

Taxon classificationAnimaliaNudibranchiaFacelinidae

Gosliner & Behrens, 1986

B5C4E855-1C29-5A26-9938-307D4815B95E

Peruvian specimen photographs: Uribe et al. (2013) , Nakamura (2007)

###### Habitat.

Benthic.

###### Depth.

3–8 m (Nakamura 2006).

###### Type material.

Holotype CASIZ 059589—Punta la Gringa, Baja California (Gosliner and Behrens 1986).

###### Distribution.

Eastern Pacific, from Baja California (Mexico, 28°N) to the northern coast of Peru (04°S) (Nakamura 2006).

###### Sampling/reporting sites.

In Peru, it was reported in Playa Las Pocitas (Mancora, Piura, 04°06'S) (Nakamura 2006) and Playa El Rubio (Tumbes) (Uribe et al. 2013, based on a personal communication with Sandra Millen). It was also reported in Panama (Camacho-García et al. 2005) and Mexico (Baja California) (Gosliner and Behrens 1986).

**Family Glaucidae Gray, 1827**

##### ﻿ Glaucus atlanticus

Taxon classificationAnimaliaNudibranchiaGlaucidae

Forster, 1777

0C1D9900-B802-556D-A0E8-C4B9C0BCC429

Peruvian specimen photographs: Uribe et al. (2013)

###### Habitat.

Pelagic.

###### Depth.

Neustonic (Churchill et al. 2014b).

###### Type material.

Not available.

###### Distribution.

Cosmopolitan and circumtropical (Churchill et al. 2014b; Thompson and McFarlane 1967).

###### Sampling/reporting sites.

Off the northern coast of Chile (Schrödl 2003). On the coast of El Salvador (13°N) (Segovia and López 2015). In Peru, it was mentioned by Paredes et al. (1999) and Ramírez et al. (2003) based on the records of d’Orbigny (1854) in Callao (10°15'S). Recently reported in Isla Santa, Ancash (09°01’S) by Uribe et al. (2013).

###### Remarks.

Included in Paredes et al. (1999), probably based on a personal communication with Sandra Millen. The records of *Glaucusdistichoicus* d’Orbigny, 1837 (d’Orbigny 1854; Dall 1909; Paredes et al. 1999; Ramírez et al. 2003) do not have enough evidence to formalize the species within the genus *Glaucus* and could refer to *G.atlanticus*.

**Superfamily Arminoidea Iredale & O’Donoghue, 1923 (1841)**

**Family Arminidae Iredale & O’Donoghue, 1923 (1841)**

##### ﻿ Armina californica

Taxon classificationAnimaliaNudibranchiaArminidae

(J.G. Cooper, 1863)

3B56C288-1ACD-52C6-BF9B-6C3EC0CCF03E

###### Habitat.

Benthic.

###### Depth.

11–268 m (Báez et al. 2011).

###### Type material.

Not available.

###### Distribution.

Eastern Pacific, from the Gulf of Alaska (Báez et al. 2011) to Piura (northern coast of Peru) (Dall 1909; Baez et al. 2011).

###### Sampling/reporting sites.

In Peru, it was reported in Paita (Piura) (Dall 1909). Baez reported a Peruvian specimen (USNM 805043 South Pacific Ocean, Peru) but lacks geographic reference. It was also reported in Alaska (Central Aleutian Islands), Canada (Scott Islands), United States (California), Mexico (Isla Tortuga, Baja California, Socorro Island), and Panama (Islas Ladrones) (see Báez et al. 2011).

###### Remarks.

In Peru, it was initially reported in Paita (Piura) (Dall 1909) under the name Pleurophyllidia (Diphyllidia) cuvierii d’Orbigny, 1837 and later considered as *Arminacuvieri* (d’Orbigny, 1837). However, *D.cuvieri* currently corresponds to *Arminatigrina* Rafinesque, 1814, a species from the Mediterranean Sea (Thompson et al. 1990). Nakamura (2006), referring to Dall (1909), listed *A.californica* as *A.cuvieri*. Báez et al. (2011) examined specimens of *A.cuvieri* and discovered an identification error, as it was actually *A.californica*. This correction is adopted in Uribe et al. (2013).

**Superfamily Dendronotoidea Allman, 1845**

**Family Dendronotidae Allman, 1845**

##### ﻿ Dendronotus cf. venustus

Taxon classificationAnimaliaNudibranchiaDendronotidae

MacFarland, 1966

566F783B-64F7-5E74-B048-3246C0041682

Peruvian specimen photograph: Uribe et al. (2013) Common name: Branched Dendronotid

###### Habitat.

Benthic.

###### Depth.

5–20 m (Korshunova et al. 2020).

###### Type material.

Not available.

###### Distribution.

From Alaska (Stout et al. 2010) to Coliumo Bay (Chile, 36°32'S) (Schrödl 2003).

###### Sampling/reporting sites.

in Peru, it was reported in Pucusana (12°25'S) as Dendronotuscf.venustus (Uribe et al. 2013). It was also reported in Alaska (Stout et al. 2010), United States (Morro Bay and Crescent City, California) (MacFarland 1966), and Chile (Coliumo Bay, 36°32'S) (Schrödl 2003).

###### Remarks.

Paredes et al. (1999) listed this species as *Dendronotusfrondosus* (Ascanius, 1774), a North Atlantic species (Ekimova et al. 2015), based on personal communication with Sandra Millen. *Dendronotusfrondosus* was confirmed to have morphological and molecular differences with *Dendronotusvenustus* (Stout et al. 2010; Ekimova et al. 2015) that is exclusive to the Pacific.

**Family Dotidae Gray, 1853**

##### ﻿ Doto uva

Taxon classificationAnimaliaNudibranchiaDotidae

Er. Marcus, 1955

14F55CD0-1710-5D57-B74E-DBD045533397

Peruvian specimen photographs: Uribe et al. (2013) , Schrödl and Hooker (2014) Common name: Grape-cluster Nudibranch

###### Habitat.

Benthic.

###### Depth.

0–15 m (Schrödl and Hooker 2014).

###### Type material.

Not available.

###### Distribution.

Amphi-South American. On the Pacific side of South America, it extends from Callao (Peru, 12°S) to Comau Fjord (Chile, 42°S) (Schrödl et al. 2005). On the Atlantic side it ranges from San Matías gulf (Argentina, 41°26'S) (Cetra and Roche 2023) to Sao Paulo (Brazil, 21°S) (Marcus 1959).

###### Sampling/reporting sites.

In Peru, it was reported in Callao (12°S), San Juan de Marcona (15°21′S), Islas Ballestas (13°44′S) (Schrödl and Hooker 2014) and Independencia Bay (14°14'S) (Uribe et al. 2013). In Chile, it was collected in Tocopilla (22°05'S), La Herradura (Coquimbo, 29°59′S), Tongoy (Coquimbo, 30°15′S) (Fischer and Cervera 2005b), Coliumo Bay (36°32'S) (Schrödl 2003), Canal de Calbuco (41°45'S) (Marcus 1959), and in Comau Fjord (42°22′S) (Schrödl et al. 2005). In Argentina, it was collected in San Matías Gulf (Patagonia, 41°30′S) (Cetra and Roche 2023). In Brazil, it was reported in Sao Paulo (21°21'S) (Marcus 1959).

###### Remarks.

Molecular studies are needed to clarify the genetic identities of the populations on both sides of South America (Schrödl 2003; Uribe et al. 2013).

**Family Hancockiidae MacFarland, 1923**

##### ﻿ Hancockia schoeferti

Taxon classificationAnimaliaNudibranchiaHancockiidae

Schrödl, 1999

F6A322B3-0DFB-5CBA-95E2-D234241F1162

Peruvian specimen photograph: Schrödl and Hooker (2014)

###### Habitat.

Benthic.

###### Depth.

0–3 m (Schrödl and Hooker 2014).

###### Type material.

Holotype ZSM Moll 19983471—Coliumo Bay (36°32'S), Chile (Schrödl 1999a).

###### Distribution.

San Juan de Marcona, Peru (15°21′S) (Schrödl and Hooker 2014) and southern Chile (37–43°S) (Schrödl 2009).

###### Sampling/reporting sites.

In Peru, it was reported for the first time in San Juan de Marcona (Ica, 15°21′S) (Schrödl and Hooker 2014). In Chile, it was reported in Coliumo Bay (36°32'S) and Queule (39°23'S) (Schrödl 1999a).

**Family Phylliroidae Menke, 1830**

##### ﻿ Cephalopyge trematoides

Taxon classificationAnimaliaNudibranchiaPhylliroidae

(Chun, 1889)

0A017E29-E5EE-54A1-B5DC-EA82C0DC5642

Peruvian specimen photograph: Quesquen (2017)

###### Habitat.

Pelagic.

###### Depth.

40 m (Fernández-Alamo 1997).

###### Type material.

Not available.

###### Distribution.

Circumtropical (van der Spoel et al. 1997).

###### Sampling/reporting sites.

In Peru, it was reported in Piura (Quesquen 2017). It was also reported in the Canary Islands and Cape Verde (Hernández et al. 2001, 2017), New South Wales (Steinberg 1956), Gulf of California (Fernández-Alamo 1997), and central and northern Chilean coast (Tokioka 1963).

###### Remarks.

Originally described as *Phylliroetrematoides* Chun, 1889. The samples described in Quesquen (2017) were reported in grey literature (Quesquen 2008) and had been previously reported by Quesquen and Guzmán (1999).

##### ﻿ Phylliroe bucephala

Taxon classificationAnimaliaNudibranchiaPhylliroidae

Lamarck, 1816

811F9E2F-C14C-5B59-B25E-2512ED5151BA

Peruvian specimen photograph: Quesquen (2017)

###### Habitat.

Pelagic.

###### Depth.

40–60 m (Fernández-Alamo 1997).

###### Type material.

Not available.

###### Distribution.

Circumtropical (van der Spoel et al. 1997).

###### Sampling/reporting sites.

In Peru, it was reported in Tumbes and Piura (Quesquen 2017). It was also reported in the Canary Islands (Hernández and Jiménez 1996), off the coasts of Florida and Bermuda (Abbott 1974), in northeastern Atlantic waters near the African coast (van der Spoel 1970), and in the western Atlantic Ocean (Spencer et al. 2009). In the Mediterranean Sea there are reports from France and Syria (Durgham et al. 2016; Durgham and Ikhtiyar 2020; Pruvot-Fol 1954). Recorded south of the Pacific Ocean in Australia and New Zealand (Powell 1979; Spencer and Willan 1995). In the Indo-Pacific it has been reported from Vietnam (Sachidhanandam et al. 2000).

**Family Cuthonidae Odhner, 1934**

##### ﻿ Cuthona

Taxon classificationAnimaliaNudibranchiaCuthonidae

sp.

C4B84399-718B-5BD9-BF1C-51CFADD51D91

Peruvian specimen photograph: Nakamura (2006)

###### Habitat.

Benthic.

###### Depth.

5–7 m (Nakamura 2006).

###### Distribution.

Northern coast of Peru.

###### Sampling/reporting sites.

Cancas (Tumbes, 03°56'S) (Nakamura 2006).

###### Remarks.

Description provides a length of 5 mm, body completely white, including rhinophores and oral tentacles with a translucent base. In addition, the specimen had dark, reddish-brown cerata without the white tip, which would differentiate it from other species of the genus (Nakamura 2006).

**Family Fionidae Gray, 1857**

##### ﻿ Fiona pinnata

Taxon classificationAnimaliaNudibranchiaFionidae

(Eschscholtz, 1831)

01304DAC-3D4E-5758-8358-91CE139D395E

###### Habitat.

Pelagic.

###### Depth.

Neustonic (Willan 1979).

###### Type material.

Not available.

###### Distribution.

Cosmopolitan (Gosliner 1987; Schmekel and Portmann 1982).

###### Sampling/reporting sites.

In Peru, it was reported in Lima (d’Orbigny 1835–1846; Álamo and Valdivieso 1997; Dall 1909). It was also reported in Chile (Mehuín, Valdivia, Juan Fernández Islands and Talcahuano) (Fischer and Cervera 2005b; Bergh 1898).

###### Remarks.

Originally named *Eolidiapinnata*Eschscholtz 1831 (type specimen from Alaska), until its current designation as *F.pinnata*; it is a species with a wide latitudinal range (Trickey 2013).

**Family Flabellinidae Bergh, 1889**

##### ﻿ Kynaria cynara

Taxon classificationAnimaliaNudibranchiaFlabellinidae

(Ev. Marcus & Er. Marcus, 1967)

8483A7DF-4877-50AD-9B3F-CA132DC1F51C

**Figure 6.**
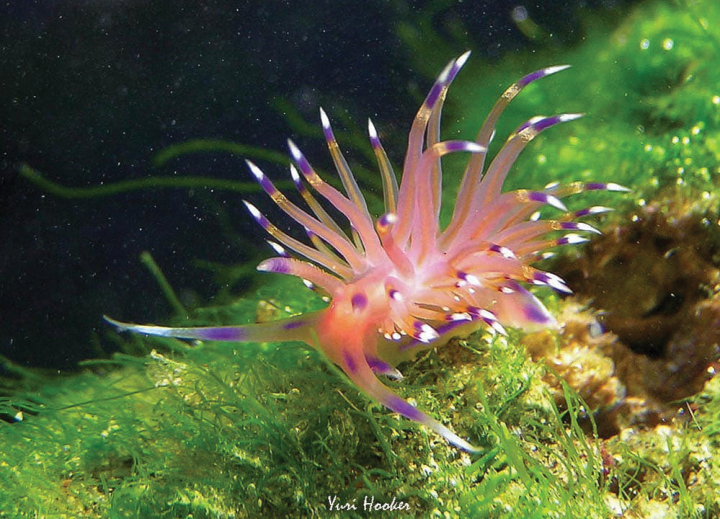
*Kynariacynara*, courtesy of Yuri Hooker.

Peruvian specimen photographs: Fig. 6 , Uribe et al. (2013) , Nakamura (2006) Common name: Swimming Cynara

###### Habitat.

Benthic.

###### Depth.

5–8 m (Nakamura 2006).

###### Type material.

Holotype USNM 678417—La Choya Bay (31°20′30″N, 113°38′06″W), Puerto Peñasco, Sonora, Mexico.

###### Distribution.

Eastern Pacific, from Gulf of California (Mexico, 28°N) (Millen and Hermosillo 2007) to Isla Tortuga (Peru, 09°S) (Uribe et al. 2013).

###### Sampling/reporting sites.

In Peru, it was reported in Punta Sal (03°56'S), Cancas (03°56'S), Mancora (04°6'S), Chimbote (09°4'S), and Ancash (09°S) (Nakamura 2006; Uribe et al. 2013). It was recently reported in Isla La Viuda (09°20′57″S) and Isla Tortuga (09°21′48″S) in Ancash (Uribe et al. 2019). It was also reported in Mexico (Gulf of California) (Millen and Hermosillo 2007) and in other several points (occurrences) such as Mexico (Guerrero), Costa Rica (Tamarindo Bay, Parque Nacional Santa Rosa), Ecuador (Salinas) (iNaturalist 2023b).

###### Remarks.

The species was originally described as *Coryphellacynara* Ev. Marcus & Er. Marcus, 1967 and reported along the Peruvian coast as *Flabellinacynara* (Nakamura 2006; Uribe et al. 2013, 2019). These designations are currently invalid and have been replaced by *Kynariacynara* (Korshunova et al. 2017).

##### ﻿ Coryphellina cerverai

Taxon classificationAnimaliaNudibranchiaFlabellinidae

(M. A. Fischer, van der Velde & Roubos, 2007)

6A233EB6-E5E3-5B27-9D2D-19A61DCEA70E

Peruvian specimen photograph: Schrödl and Hooker (2014)

###### Habitat.

Benthic.

###### Depth.

0–10 m (Schrödl and Hooker 2014).

###### Type material.

Holotype RMNH Moll. 98130—La Herradura, Coquimbo (29°58'S, 071°22'W), Chile.

###### Distribution.

From Sechura Bay (Peru, 05°49'S) to Coliumo Bay (Chile, 36°32'S).

###### Sampling/reporting sites.

In Peru, it was reported in Sechura Bay (05°49'S) (Schrödl and Hooker 2014), Ancon (Lima, 11°47'S) and Pucusana (Lima, Peru, 12°25'S) (Schrödl 1996a). In Chile, it was reported in La Herradura (Coquimbo, 29°59′S) (Fischer et al. 2007), Peñón de Vida Marina (Montemar, Viña del Mar, 32°57'26.8"S) (Molina 2021), Coliumo Bay (36°32'S) (Schrödl 1996a).

###### Remarks.

The species was first reported as *Flabellina* sp. 2 (Schrödl 1996a) and then as *Flabellinacerverai* van der Velde & Roubos, 2007 (Fischer et al. 2007; Schrödl and Hooker 2014).

**Superfamily Proctonotoidea Gray, 1853**

**Family Janolidae Pruvot-Fol, 1933**

##### ﻿ Janolus rebeccae

Taxon classificationAnimaliaNudibranchiaJanolidae

Schrödl, 1996

4755933A-17E9-5872-BC65-FDFAC83A16D1

**Figure 7.**
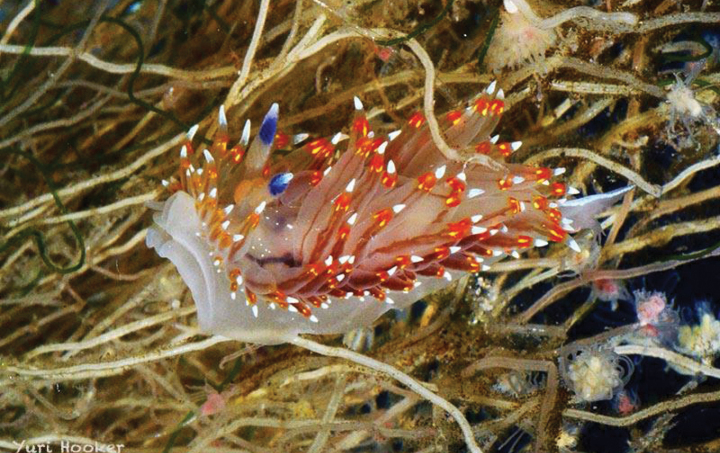
*Janolusrebeccae*, courtesy of Yuri Hooker.

Peruvian specimen photographs: Fig. 7 , Schrödl and Hooker (2014)

###### Habitat.

Benthic.

###### Depth.

2–12 m (Schrödl 1996a, b; Schrödl and Hooker 2014).

###### Type material.

Holotype ZSM 19960557—English Bay (27°07'S, 070°53'W), Chile.

###### Distribution.

From Sechura Bay (Peru, 05°49'S) (Schrödl and Hooker 2014) to English Bay (Chile, 27°07′51″S) (Schrödl 1996b, 2003).

###### Sampling/reporting sites.

In Peru, it was reported in Sechura Bay (05°49'S) and Paracas (13°43'S) (Schrödl and Hooker 2014). In Chile, it was reported in Juan Lopez (23°30'S) and English Bay (27°07'S) (Schrödl 1996a, 1996b).

**Superfamily Tritonioidea Lamarck, 1809**

**Family Tritoniidae Lamarck, 1809**

##### ﻿ Tritonia

Taxon classificationAnimaliaNudibranchiaTritoniidae

sp.

0EA42077-660A-5AFD-B804-480A9B51595C

**Figure 8.**
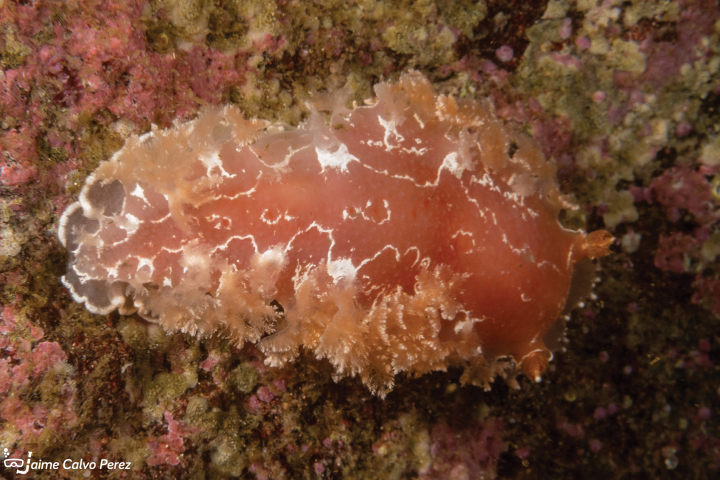
*Tritonia* sp., courtesy of Jaime Calvo-Pérez.

Peruvian specimen photographs: Fig. 8 , Uribe et al. (2013) , Uribe et al. (2019)

###### Habitat.

Benthic.

###### Depth.

5–15 m (Uribe et al. 2013).

###### Distribution.

From Foca Island (Piura, Peru, 05°12'S) to Punta Picata (Tacna, Peru) (Uribe et al. 2013).

###### Sampling/reporting sites.

In Peru, it was reported in Foca Island (Piura, 05°12'S), Santa Island (09°01'S), Ferrol Bay (Chimbote, 09°06'S), Punta El Huaro (Casma, Ancash, 09°37’S), La Gramita (Casma, Ancash, 09°43'S), Punta Patillos (Huarmey, 09°53'S), Punta Colorado (Huarmey, Ancash, 10°29′S), Pucusana (Lima, 12°25'S), Isla Asia (Lima, 12°47'S), Isla La Vieja (Independencia Bay, Pisco, Ica, 14°16′S) and Punta Picata (Tacna, 17°52'S) (Uribe et al. 2013).

###### Remarks.

It bears resemblance to *Tritoniaodhneri* (common in Chile) in terms of its external morphology, while displaying similarities to *Tritoniafestiva* (found in Alaska, Baja California, and Japan; Uribe et al. 2013). Anatomical and molecular analyses are necessary to describe this species (Uribe et al. 2013).

#### ﻿Suborder Doridina

**Superfamily Chromodoridoidea Bergh, 1891**

**Family Chromodorididae Bergh, 1891**

##### ﻿ Tyrinna delicata

Taxon classificationAnimaliaNudibranchiaChromodorididae

(Abraham, 1877)

3E4FD79E-4280-5E8A-9EAF-0A13613E0569

**Figure 9.**
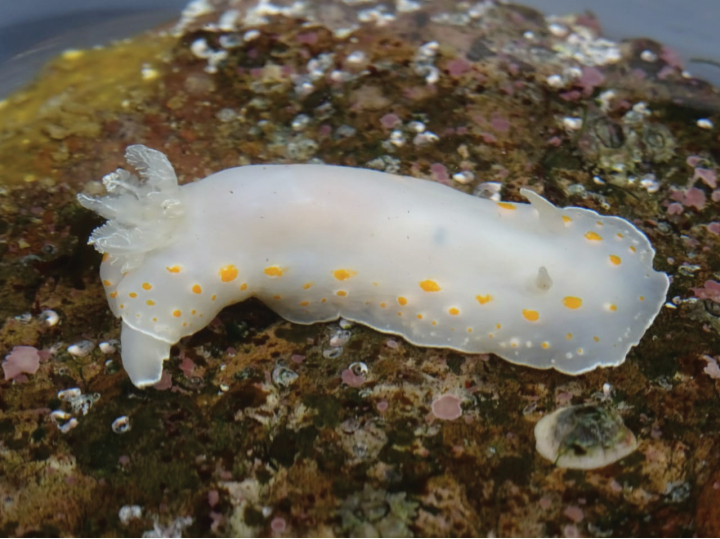
*Tyrinnadelicata*, courtesy of Fabián Avilés.

Peruvian specimen photographs: Fig. 9 , Uribe et al. (2013)

###### Habitat.

Benthic.

###### Depth.

0–22 m (Uribe et al. 2013).

###### Type material.

Holotype NHMUK 1995038—Chiloé Island, Chile.

###### Distribution.

Amphi-South American. From Pucusana (Lima, Peru, 12°25'S) (Fabián Avilés pers. comm.) to Strait of Magellan (Chile, 53°S) and Peninsula Valdés (Argentina, 42°S) (Schrödl and Millen 2001; Uribe et al. 2013; Araya and Valdés 2016).

###### Sampling/reporting sites.

In Peru, two specimens were collected (MHNURP, specimens currently lost) in Playa Las Ninfas (Pucusana, Lima, 12°28'49"S) on 23 October 2019, at 1.5–2.0 m depth, 55–60 mm length (Fig. 9) (Fabián Avilés pers. comm.). In Peru, this species was also reported in San Juan de Marcona (Ica, 15°21′S) and Isla Blanca (Arequipa, 17°00'S) (Uribe et al. 2013). In Chile, it was reported in Caleta Obispito (Caldera, Atacama, 26°45'51"S) (Araya and Valdés 2016) and in several points (occurrences) such as Reserva Nacional Pingüino de Humboldt (29°15′S), Playa El Francés (Coquimbo, 30°05'S), Pichicuy (32°20′S), Caleta Cocholgüe-Tomé (36°35'S), Caleta Chome (Península de Hualpén, 36°48'S), Caleta Chaihuín (39°56'S), Faro San Isidro (Strait of Magellan, 53°28′S) (iNaturalist 2023a). It was also reported in San Juan Fernández Islands (33°38′S) (Araya and Valdés 2016). In Argentina it was observed (occurrences) in Baliza Davison (Tierra del Fuego, 54°56'S), Grand Jason (Jason Islands, 51°04'S), La Tranquera (46°02'S), Golfo San Jorge (46°00'S), Punta del Marqués (Rada Tilly, 45°57'S), Parque Interjurisdiccional Marino Costero Patagonia Austral (PIMCPA, 45°02'S), Camarones Bay (44°46'S), Golfo Nuevo (42°47'S), and Golfo San José (42°24'S) (iNaturalist 2023c).

###### Remarks.

This species was originally identified as *Tyrinnanobilis* Bergh, 1898, a name that is currently not accepted.

##### ﻿ Tyrinna evelinae

Taxon classificationAnimaliaNudibranchiaChromodorididae

(Er. Marcus, 1958)

24BB42FD-73D8-517A-A2CF-4B6A7478975C

###### Habitat.

Benthic.

###### Depth.

0–5 m (Welch 2010).

###### Type material.

Not available.

###### Distribution.

Amphi-American and West Africa.

###### Sampling/reporting sites.

In Peru, it was reported in El Rubio (Tumbes, 03°52'S) (Schrödl and Millen 2001). It was also reported in Mexico (Gulf of California, 28°N), Jamaica (Schrödl and Millen 2001). In the Atlantic, it has been sampled in the Gulf of Mexico (de la Cruz-Francisco et al. 2017), Panama (Goodheart et al. 2016), Brazil (Marcus 1958), Ghana, and Cape Verde (Camacho-García et al. 2005).

###### Remarks.

It was initially listed by Paredes et al. (1999). According to Uribe et al. (2013) molecular studies are necessary to confirm the consistency of reports of *T.evelinae* in both the Atlantic and Pacific populations that are morphologically difficult to distinguish (Valdés et al. 2006).

##### ﻿ Felimare agassizii

Taxon classificationAnimaliaNudibranchiaChromodorididae

(Bergh, 1894)

6F4E295C-73D7-5AF3-B295-D45EBC94BD2D

Peruvian specimen photographs: Uribe et al. (2013) , Nakamura (2007) Common name: Agassiz’s Chromodorid

###### Habitat.

Benthic.

###### Depth.

7–8 m (Nakamura 2006).

###### Type material.

Not available.

###### Distribution.

From the Gulf of California to the coast of northern Peru.

###### Sampling/reporting sites.

In Peru, it was reported in Cancas (Tumbes, 03°56'S) (Nakamura 2006) and Lobos de Tierra Island (Lambayeque, 06°25′S) (Uribe et al. 2013). It was also reported in Mexico (Gulf of California, 28°N), Ecuador (Galapagos Islands, 0°S), Colombia (Malpelo Island, 04°N) (Behrens and Hermosillo 2005).

###### Remarks.

Originally described as *Chromodorisagassizii* Bergh, 1894 and reported in Peruvian waters as *Hypselodorisagassizii* by Nakamura (2006).

##### ﻿ Felimare sechurana

Taxon classificationAnimaliaNudibranchiaChromodorididae

Hoover, Padula, Schrödl, Hooker & Valdés, 2017

11680501-5808-5366-8315-875728BCC39A

**Figure 10.**
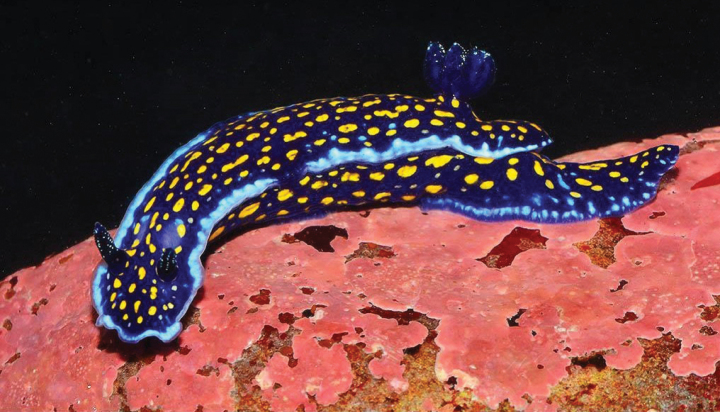
*Felimaresechurana*, courtesy of Yuri Hooker.

Peruvian specimen photographs: Fig. 10 , Hoover et al. (2017)

###### Habitat.

Benthic.

###### Depth.

6–15 m (Hoover et al. 2017; Bravo et al. 2020)

###### Type material.

Holotype CZA 402—Foca Island (05°12'13.8"S, 81°12'38.0"W), Piura, Peru.

###### Distribution.

Northern coast of Peru’s transition zone.

###### Sampling/reporting sites.

In Peru, it was reported in Punta Veleros (Los Organos, Piura, 04°10'28.7"S) (Zavala, 2022), Quebrada Verde (Piura, 04°13'34.8"S), Foca Island (Piura, 05°12'13.8"S) (Hoover et al. 2017), and Lobos de Afuera Islands (Lambayeque, 06°56′S) (Bravo et al. 2020).

###### Remarks.

This species is endemic to the northern coast of Peru. It was initially reported as *Felimareghiselini* (Bertsch 1978) by Thompson (2006) but later corrected by Hoover et al. (2017) and described as a new species.

##### ﻿ Felimida baumanni

Taxon classificationAnimaliaNudibranchiaChromodorididae

(Bertsch, 1970)

2ACCD989-D02E-5534-B1A7-EFF3D37C99B5

**Figure 11.**
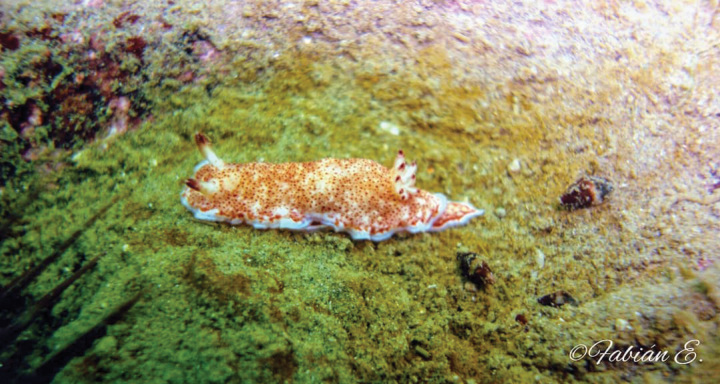
*Felimidabaumanni*, courtesy of Fabián Encinas.

Peruvian specimen photograph: Fig. 11 Common name: Baumann’s Chromodorid

###### Habitat.

Benthic.

###### Depth.

5–8 m (Nakamura 2006).

###### Type material.

Not available.

###### Distribution.

Eastern Pacific, from Gulf of California (28°N) to Cancas (Tumbes, Peru) (Nakamura 2006).

###### Sampling/reporting sites.

In Peru, it was reported in Cancas (Tumbes, 03°56'S) (Nakamura 2006). It was also reported in Mexico (Gulf of California, 28°N), Panama (08°N), Ecuador (Galapagos Islands, 00°S) and Colombia (Malpelo Islands, 04°N) (Behrens and Hermosillo 2005).

###### Remarks.

The species was originally reported as *Chromodorisbaumanni* Bertsch, 1970 in the Eastern Pacific (Rudman 1983). Years later, Gosliner et al. (2004) discussed the anatomical characteristics of this species to be like those attributed to the genus *Glossodoris*, suggesting a reclassification. Finally, the phylogenetic study by Johnson and Gosliner (2012) defined its new classification as part of the genus *Felimida*.

**Superfamily Doridoidea Rafinesque, 1815**

**Family Discodorididae Bergh, 1891**

##### ﻿ Baptodoris peruviana

Taxon classificationAnimaliaNudibranchiaDiscodorididae

(d’Orbigny, 1837)

EF0FF17D-BA29-500D-9E21-5325D909064D

Peruvian specimen photographs: Uribe et al. (2013) , Schrödl and Hooker (2014)

###### Habitat.

Benthic.

###### Depth.

4–15 m (Schrödl and Hooker 2014).

###### Type material.

Holotype ZMB 50748—Isla de Pajargo (Pájaros), Chile (as *Platydorispunctatella* Bergh, 1898), poorly preserved. Neotype SSUC 6977 (*Dorisperuviana*), Iquique (Chile, 20°12'S), February 1965, undissected specimen (Fischer and Cervera 2005a).

###### Distribution.

From San Lorenzo Island (Callao, Peru, 12°S) (d’Orbigny 1835–1846) to Los Molles (Valparaíso, 32°15'S) (Fischer and Cervera 2005).

###### Sampling/reporting sites.

In Peru it was reported for first time as *Doriopsisperuviana* in San Lorenzo Island (12°05′) by d’Orbigny (1835–1846). It was also reported in Callao (12°S, as *Dorisperuviana*, Dall 1909), Pucusana (Lima, 12°28'S, as *Platydorispunctatella*, Schrödl 1996a), Tortugas Bay (Ancash, 09°21′S), Independencia Bay (Ica, 14°14'S), and San Juan de Marcona (Ica, 15°21′S) (Uribe et al. 2013) In Chile, it was collected in Iquique (20°12'S), Mejillones (23°20'S), La Portada (Antofagasta, 23°39'S), and Los Molles (Valparaíso, 32°15'S) (Fischer and Cervera 2005a).

###### Remarks.

Initially reported as *Dorisperuviana* d’Orbigny 1836, transferred to *Platydoris* Bergh, 1877 by Schrödl (2003), who also included Bergh’s (1898) description of *P.punctatella* as a junior synonym. Dorgan et al. (2002) ruled out that these reports were about a species belonging to *Platydoris*, based on a photograph of a live specimen (Schrödl 1996a). Based on the description of the radular teeth, this species was transferred from *Platydoris* to the genus *Baptodoris* (Fischer and Cervera 2005a). Regarding its northernmost distribution, Fischer and Cervera (2005a) considered the reports from Tagus Cove (Albermarle, Galapagos Islands) (Pilsbry and Vanatta 1902) as doubtful.

##### ﻿ Diaulula variolata

Taxon classificationAnimaliaNudibranchiaDiscodorididae

(d’Orbigny, 1837)

B12BD633-AC07-5A4D-B926-7EAD5409B13F

**Figure 12.**
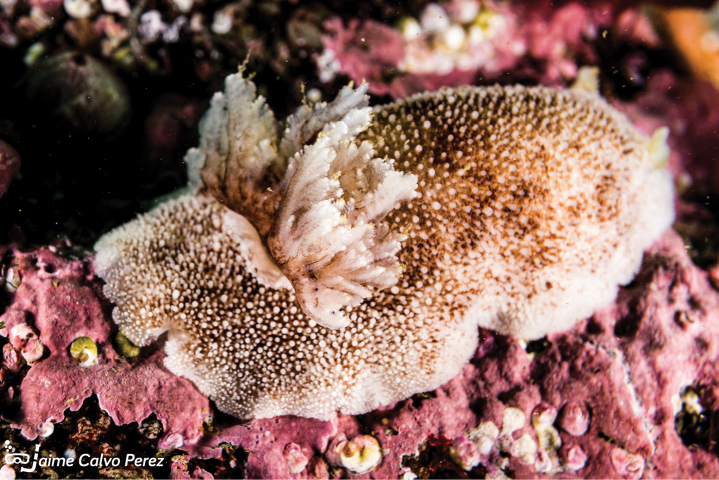
*Diaululavariolata*, courtesy of Jaime Calvo-Pérez.

Peruvian specimen photographs: Fig. 12 , Schrödl and Hooker (2014)

###### Habitat.

Benthic.

###### Depth.

2–15 m (Schrödl and Hooker 2014).

###### Type material.

Not available.

###### Distribution.

From Pucusana (Lima, Peru, 12°28'S) (Guzman 2018b) to Punta Hualpén (Concepción, Chile, 36°44'S) (Marcus 1959).

###### Sampling/reporting sites.

In Peru, it was reported in Pucusana (Lima, 12°28'S) (Guzman 2018b), El Chaco (Ica, 13°49'S), Caleta Atenas (Ica, 13°49'S), Independencia Bay (Ica, 14°14'S), San Juan de Marcona (Ica, 15°21′S) (Uribe et al. 2013), Dos Playas (Arequipa, 17°00'S) and Playa Calera (Arequipa, 17°15'S) (Tejada-Pérez et al. 2018; Valdivia-Chavez et al. 2020), Caleta La Huata (Camaná, Arequipa, 16°50'S) (Zanabria 2020a), Terminal Portuario Matarani (Islay, Arequipa, 16°59'S) (Zanabria 2020b), and Playa Jaboncillo (Ilo, Moquegua, 17°59'S) (Zanabria 2020c).

In Chile, it was reported in Arica (18°26'S) (Schrödl 2003), Coquimbo Bay (29°57'S) (Bergh 1898; Valdés and Muniaín 2002), English Bay-Guanaqueros-Los Hornos (30°10'S), El Tabo (Valparaíso, 33°27'S) (Fischer and Cervera 2005b), Coliumo Bay (36°32'S) (Schrödl 1996a, 1997), San Vicente Bay (36°44'S), and Punta Hualpén (Concepción, 36°44'S) (Marcus 1959). Within its Chilean distribution, several other recent observations (occurrences) have been reported (iNaturalist 2023d).

###### Remarks.

This species had not been recorded outside Chile (Fischer and Cervera 2005b) until the first reports from Peru (Uribe et al. 2013). The specimen collected in Bernardo O’Higgins National Park (Chilean fjord region, 51°S) (Aldea et al. 2011) needs confirmation (Uribe et al. 2013).

##### ﻿ Diaulula punctuolata

Taxon classificationAnimaliaNudibranchiaDiscodorididae

(d’Orbigny, 1837)

49938861-0205-569C-8C63-8759D68524CD

###### Habitat.

Benthic.

###### Depth.

0–7 m.

###### Type material.

ZSM Moll 20040984—Ipún Island (44°33'S, 74°48'W), Aysén, Chile.

###### Distribution.

Amphi-South American. It is frequently found on the Magellanic coasts of Chile and Argentina.

###### Sampling/reporting sites.

In Peru, it was collected in Callao (12°S) (Dall 1909; Schrödl 2003). In Chile, it was collected in Lota (37°05′S), Lacuy Peninsula (Greater Island of Chiloé, 41°49′S) (Valdés and Muniaín 2002), Ipún Island (Chonos Archipelago, 44°33'S) (Schrödl and Grau 2006) and Strait of Magellan (53°35'S) (Roche et al. 2023). In Argentina, it was collected in San Matías Gulf (41°30′S) (Roche et al. 2023; Cetra 2019), Gulf Nuevo (42°42′S) (Valdés and Muniaín 2002), Peninsula Valdés (42°30'S), Punta Pardelas (42°36′S), Puerto Madryn (42°46′S) (Roche et al. 2023; Cetra 2019), Comodoro Rivadavia (45°51'S) (Valdés and Muniaín 2002), and Tierra del Fuego (54°21′S) (Roche et al. 2023).

###### Remarks.

This species was listed as *Anisodorispunctuolata* (d’Orbigny, 1836) and *Dorispunctuolata* d’Orbigny, 1837 in previous Peruvian articles listing nudibranch species. Both names are currently not accepted.

##### ﻿ Rostanga pulchra

Taxon classificationAnimaliaNudibranchiaDiscodorididae

MacFarland, 1905

73AD824C-BCA7-58E8-9143-926DFCB7CE2C

Peruvian specimen photographs: Schrödl and Hooker (2014) Common name: Red Sponge Dorid

###### Habitat.

Benthic.

###### Depth.

6–12 m (Schrödl and Hooker 2014).

###### Type material.

Holotype USNM 181292—Monterey Bay, California, United States.

###### Distribution.

This species presents a bipolar distribution in the Eastern Pacific and an amphi-South American distribution (Schrödl 2003; Schrödl and Grau 2006).

###### Sampling/reporting sites.

In Peru it was considered a predicted species (Uribe et al. 2013) until the first specimen was finally reported in Punta San Juan (Marcona, Ica, 15°22′S) (Schrödl and Hooker 2014). It has been also reported in Alaska (Point Craven) (Lee and Foster 1985), Mexico Los Angeles Bay) (Lance 1966), Chile (Coliumo Bay, Playa Brava and Chonos archipelago) (Marcus 1959; Schrödl 2003; Schrödl and Grau 2006), and Argentina (Camarones Bay) (Marcus and Marcus 1969).

###### Remarks.

Its distribution in Peruvian Waters was not certain; however, it was listed by Álamo and Valdivieso (1997) and Paredes et al. (1999). *Rostangapulchra* is the only species of the genus in Peruvian and Chilean waters. Schrödl and Hooker (2014) mentioned that populations in the northern and southern hemispheres are likely to be distinct species based on preliminary unpublished molecular data.

**Family Dorididae Rafinesque, 1815**

##### ﻿ Doris fontainii

Taxon classificationAnimaliaNudibranchiaDorididae

d’Orbigny, 1837

713EB458-7741-55A2-9471-8B7986138C6B

**Figure 13.**
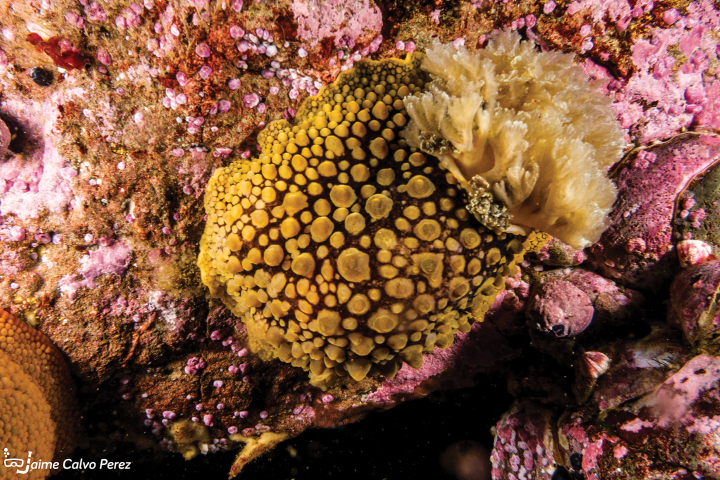
*Dorisfontainii*, courtesy of Jaime Calvo-Pérez.

Peruvian specimen photographs: Fig. 13

###### Habitat.

Benthic.

###### Depth.

8–17 m (Schrödl and Hooker 2014).

###### Type material.

Holotype ZSM 19983417. Coliumo Bay, Chile (36°32'S, 72°57'W).

###### Distribution.

Amphi-South American.

###### Sampling/reporting sites.

In Peru it was reported in Independencia Bay (14°14'S) ([Bibr B133][Bibr B142]; Rudman 2002a, 2002b; Uribe et al. 2013), San Fernando National Reserve (14°58′S) (Ampuero 2010), and recently in Arequipa (15–17°S) (Valdivia-Chavez et al. 2020). Other reports (occurrences) for this species in the Peruvian coast are Pucusana, Ilo, Marcona, Pisco, Islay and Camaná, (iNaturalist 2023e). In Chile it was reported in Arica (18°28'S), Coquimbo (29°57'S), Dichato (36°32'S), Puerto Montt (41°27'S) (Valdés and Muniaín 2002; Schrödl 2003), and the Chonos Archipelago (45°08′S) (Schrödl and Grau 2006). In Argentina, it was collected from 37°50'S to Rada Tilly (45°55'S) (Valdés and Muniaín 2002).

###### Remarks.

Erroneously named as *Dorisfontainei* in previous articles.

**Superfamily Onchidoridoidea Gray, 1827**

**Family Corambidae Bergh, 1871**

##### ﻿ Corambe lucea

Taxon classificationAnimaliaNudibranchiaCorambidae

Er. Marcus, 1959

2513C965-0E45-545D-B875-2FAFCA88644A

Peruvian specimen photograph: Schrödl and Hooker (2014)

###### Habitat.

Benthic.

###### Type material.

Neotype ZSM 1912—Caleta Buena (Chile, 22°25'S, 70°15'W).

###### Depth.

0–27 m (Schrödl and Hooker 2014)

###### Distribution.

From Bayóvar (Sechura Bay, Peru, 05°49'S) (Schrödl and Hooker 2014) to Gulf of Corcovado (Chile, 42°46'50"S) (Schrödl 1996a).

###### Sampling/reporting sites.

In Peru, it was reported in Bayóvar (Sechura Bay, 05°49'S), Callao (pier of IMARPE, 12°03'59"S), Ballestas Islands (Paracas, 13°43'54"S) and San Juan de Marcona (Ica, 15°21′S) (Schrödl and Hooker 2014). In Chile, it was reported in Caleta Buena (22°25'S), Coliumo Bay (36°32'S) (Schrödl 1997), Faro Punta Corona (Chiloé, 41°50'S) (Marcus, 1959), Ancud Bay (41°52'S) (Schrödl 1996a), Comau Fjord (42°22'S) (Schrödl et al. 2005) and Gulf of Corcovado (42°46'S) (Schrödl 1996a).

###### Remarks.

It was first described as *Neocorambelucea* (Schrödl 1996a). A morphological analysis confirmed its status as a valid species (Schrödl and Wägele 2001).

##### ﻿ Corambe mancorensis

Taxon classificationAnimaliaNudibranchiaCorambidae

Martynov, Brenzinger, Hooker & Schrödl, 2011

F6BDE046-C0B9-52BC-ADDC-3E94950A56E8

Peruvian specimen photograph: Martynov et al. (2011)

###### Habitat.

Benthic.

###### Depth.

0–3 m (Martynov et al. 2011).

###### Type material.

ZSM 20080543—Mancora (04°06'36"S, 81°04'02"W), Piura, Peru.

###### Distribution.

Species only reported off the coast of Mancora (Piura, Peru) (Martynov et al. 2011).

###### Remarks.

Endemic species of northern coast of Peru.

**Family Goniodorididae H. Adams & A. Adams, 1854**

##### ﻿ Okenia luna

Taxon classificationAnimaliaNudibranchiaGoniodorididae

Millen, Schrödl, Vargas & Indacochea, 1994

DF8F066F-D853-54FB-BB8E-5C14BCF910B1

**Figure 14.**
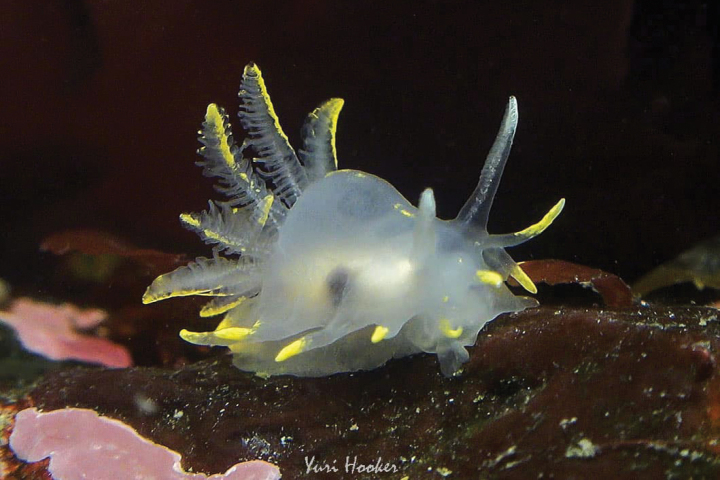
*Okenialuna*, courtesy of Yuri Hooker.

Peruvian specimen photographs: Fig. 14 , Schrödl and Hooker (2014)

###### Habitat.

Benthic.

###### Depth.

4–20 m (Schrödl 1996a).

###### Type material.

Holotype CASIZ 089293—Coliumo Bay (36°32'S, 73°57'W), north of Concepción, Chile.

###### Distribution.

From Ancon Bay (Lima, Peru) to Coliumo Bay (Chile).

###### Sampling/reporting sites.

In Peru, it was collected in Ancon Bay (Lima, 11°47'S) (Millen et al. 1994). In Chile, it was collected in Iquique (22°13'S) (Fischer 2006) and Coliumo Bay (36°32'S) (Schrödl 2003).

###### Remarks.

First record of the genus *Okenia* reported in the Southeast Pacific (Millen et al. 1994). It is distributed in Peruvian and Chilean waters.

**Superfamily Phyllidioidea Rafinesque, 1814**

**Family Dendrodorididae O’Donoghue, 1924 (1864)**

##### ﻿ Doriopsilla janaina

Taxon classificationAnimaliaNudibranchiaDendrodorididae

Er. Marcus & Ev. Marcus, 1967

D505ADCA-5ABF-55CB-9CE9-8EDAFB89BDDE

Peruvian specimen photograph: Nakamura (2007)

###### Habitat.

Benthic.

###### Depth.

0–3 m (Nakamura 2006).

###### Type material.

Holotype USNM 576269—Panama Canal (09°05′N, 79°41′W), Panama.

###### Distribution.

From the Gulf of California (28°N) to Cancas (Peru, 03°56'S).

###### Sampling/reporting sites.

In Peru, it was reported in Cancas (Tumbes, 03°56'S) (Nakamura, 2006). It was also reported in Mexico (Punta Lobos, Sonora), Panama (08°N), and Ecuador (Galapagos Islands, 00°S) (Gosliner 1991).

**Superfamily Polyceroidea Alder & Hancock, 1845**

**Family Polyceridae Alder & Hancock, 1845**

##### ﻿ Polycera priva

Taxon classificationAnimaliaNudibranchiaPolyceridae

Er. Marcus, 1959

6DC5DEFC-722A-55B3-8A85-2F0D3755670F

Peruvian specimen photographs: Schrödl and Hooker (2014)

###### Habitat.

Benthic.

###### Depth.

10 m (Schrödl 1996a).

###### Type material.

Holotype ZSM Moll. 20041005—Punta Llonco, Comau Fjord (42°22′10″S, 072°27′18″W), Chile.

###### Distribution.

From Paracas (Ica, Peru, 13°43'S) (Schrödl and Hooker 2014) to Chilean Patagonia (54°S) (Schrödl 2009; Schrödl et al. 2005).

###### Sampling/reporting sites.

In Peru, it was reported in Ballestas Islands (Lima, 13°43'S) (Schrödl and Hooker 2014). In Chile, it was reported in Gulf of Ancud (42°06′S) (Marcus 1959) and Comau Fjord (42°22′S) (Schrödl et al. 2005).

###### Remarks.

Peruvian presence of this species was surprising for the discoverers (Schrödl and Hooker 2014) since it was considered a Magellanic species, being very abundant in the fjords of southern Chile (41–44°S) (Schrödl 1996a, 1999b, 2003, 2009).

### ﻿Potentially occurring species (unconfirmed)

#### ﻿ Thecacera darwini

Taxon classificationAnimaliaNudibranchiaPolyceridae

Pruvot-Fol, 1950

ACB88EA6-D355-5BD3-8AB5-45C23727632E

##### Habitat.

Benthic.

##### Type locality.

Orange Bay (55°31'S), Nassau Bay, Chile (Valdés and Héros 1998).

##### Distribution.

From Juan López, northern coast of Chile, to Strait of Magellan (Schrödl 2003).

##### Remarks.

This species was listed by Nakamura (2006) based on Schrödl (1999b), who listed this species as present in the Peruvian province, referring to Chilean waters. Fischer and Cervera (2005b) argued against its occurrence off the Chilean coast, although they acknowledge a high likelihood of its presence in Peruvian waters. Uribe et al. (2013) and Fischer (2006) referred to Zagal and Hermosilla’s (2001) presumed finding of this species in Peru.

#### ﻿ Glaucus

Taxon classificationAnimaliaNudibranchiaPolyceridae

sp.

40D1EF73-362C-5226-A09A-34A8105DF49E

##### Habitat.

Pelagic.

##### Type material.

Not available.

##### Distribution.

Ica (Peru).

##### Sampling/reporting sites.

In Peru, Quesquen (2017) reported specimens twice off the coast of Ica in 1995 and 1998. Currently, the only valid species of the genus *Glaucus* is *G.atlanticus*, reported in Peru by Uribe et al. (2013) from Isla Santa, Ancash.

##### Remarks.

Quesquen (2017) offered a description of the specimens collected in Ica. He described a slender body that was ventrally flattened, with a small head and two cephalic tentacles. The dorsum was navy blue and green, and the ventral area was white. Additionally, the specimens had three or four branches on both sides of the body, and their body length could reach up to 43 mm. According to Valdés and Campillo's (2004) description, *G.atlanticus* is characterized by its slim and elongated body, along with a small head and sleek oral tentacles and rhinophores. The coloration of its dorsum can vary from deep blue to brown hues. It possesses up to three groups of cerata, and its ventral region exhibits a silver shade.

A confirmation of the taxonomic status of these specimens is necessary, using morphological and molecular analyses.

#### ﻿ Aeolidia campbellii

Taxon classificationAnimaliaNudibranchiaPolyceridae

(Cunningham, 1871)

44281468-98A7-5B1E-8449-9BA1E3F5F833

##### Habitat.

Benthic.

##### Type material.

ZSM 20020700 (Chile), designated as the neotype due to absence of the holotype (Kienberger et al. 2016).

##### Distribution.

Falkland Islands (50°S), Argentine and Chilean Patagonia (41°S) to Valparaiso (32°S) (Schrödl 2003). In Peru, this species was listed by Paredes et al. (1999) as *Aeolidiaserotina* Bergh, 1873 and replicated by Ramírez et al. (2003).

##### Remarks.

It was reported off the coast of Chile by Schrödl (2003) as *Aeolidiapapillosa* (Linnaeus, 1761), later reassigned to the species *A.campbellii* according to molecular studies by Kienberger et al. (2016).

#### ﻿ Gargamella immaculata

Taxon classificationAnimaliaNudibranchiaPolyceridae

Bergh, 1894

F1CD1B5B-8CA3-5B15-B071-C1C1213E0C1E

##### Habitat.

Benthic.

##### Type material.

SMNH 1015—Tierra del Fuego, Chile.

##### Distribution.

A common species on the southern coast of Chile and Argentina.

##### Sampling/reporting sites.

Cabo Metalqui, Chiloé (Fischer and Cervera 2005b; Odhner 1926), in Última Esperanza, Tierra del Fuego (Marcus 1959), in Cabo San Antonio; Cabo Delgado; Gulf of Ancud, between Isla Quenu and Calbuco; Seno Otway, Queule and Coliumo Bay (Schrödl 1996a). In Argentina, in the north (Bergh 1894; Odhner 1926), also in Argentine Patagonia and on the Burdwood Bank (Odhner 1926; Schrödl 2003).

##### Remarks.

According to Schrödl (2003), the Peruvian records of this species by Zagal and Hermosilla (2001) are doubtful. However, in that compilation and in the most up-to-date publication (Zagal and Hermosilla 2007), *Gargamellaimmaculata* is not mentioned from Peruvian waters, but as an inhabitant of the Peruvian zoogeographic province from Juan López (Atacama) to the south. The species was mistakenly included in Kentrodorididae by Schrödl (1996a), until Valdés (2002) transferred it back to the Discodorididae.

#### ﻿ Cadlina sparsa

Taxon classificationAnimaliaNudibranchiaPolyceridae

(Odhner, 1922)

A078A087-7194-5ADF-81CD-FF2D017CED01

##### Habitat.

Benthic.

##### Type locality.

Juan Fernández Islands and Desventuradas Islands, Chile (Odhner 1922).

##### Distribution.

It presents disjunct populations with a bipolar distribution in the eastern Pacific and an amphi-South American pattern.

##### Sampling/reporting sites.

In the Pacific, the northernmost location is Baja California (Behrens 1991; Jaeckle 1983) and the southernmost location is the Comau Fjord in southern Chile. In the Atlantic, it was recorded in Camarones Bay in the central region of Argentina (Schrödl 2000, 2003). In Chile, it has also been sampled in the Juan Fernández Islands (Odhner 1922), Chiloé Islands (Marcus 1959), and Coliumo Bay (Schrödl 1996a, 2003).

##### Remarks.

*Cadlinasparsa* was initially proposed as probable species in Peruvian water by Álamo and Valdivieso (1997). Subsequently, its presence was consistently mentioned in the lists compiled by Paredes et al. (1999), Ramírez et al. (2003), and Nakamura (2006). However, no actual specimens have been collected from intermediate Pacific locations, including Peru. Despite the absence of direct observations, a hypothetical distribution for Peru has been predicted through extrapolation, assuming a continuous geographic range (Uribe et al. 2013).

Studies have demonstrated that *C.sparsa* does not fall within the family Chromodorididae, as initially suggested (Johnson 2011), but rather belongs to the family Cadlinidae. However, there have been no other revisions or updates regarding its scientific name. The taxonomy of the genera involved has been thoroughly elucidated in previous literature (Schrödl 2000).

#### ﻿ Polycera cf. alabe

Taxon classificationAnimaliaNudibranchiaPolyceridae

Collier & Farmer, 1964

0974856A-FB26-5D12-ACF7-22613A38D7AD

##### Habitat.

Benthic.

##### Type material.

CASIZ 18190—Cedros Island, Baja California (28°12'13"N, 115°15'28"W), Mexico.

##### Distribution.

From Baja California (Behrens 2004; Behrens and Hermosillo 2005; Camacho-García et al. 2005), Puerto Vallarta (20°40'N) in Mexico to Costa Rica (Behrens 2004); and northern Chile with a single isolated record (Schrödl 2003).

##### Remarks.

In Peru, Paredes et al. (1999) listed this species as Polyceracf.alabe, likely based on information obtained through personal communication with Sandra Millen. Subsequently, Uribe et al. (2013) included this species in their listing, also citing personal communication with Sandra Millen, who observed this species at El Rubio (Tumbes).

#### ﻿ Phylliroe lichtensteinii

Taxon classificationAnimaliaNudibranchiaPolyceridae

Eschscholtz, 1825

B716EAC3-1207-59A6-AF3C-B6827648575C

##### Habitat.

Pelagic.

##### Type material.

Not available.

##### Distribution.

Cosmopolitan (Padula 2015).

##### Sampling/reporting sites.

Espiritu Santo, southeastern Brazil (Ralph 1959).

##### Remarks.

For Peru, the species was listed in Ramírez et al. (2003). There are no reports of collections of this species in Peruvian waters.

#### ﻿ Itaxia falklandica

Taxon classificationAnimaliaNudibranchiaPolyceridae

(Eliot, 1907)

0BD6E35D-1D30-5CA7-8B23-2B95825C8075

##### Habitat.

Benthic.

##### Depth.

1–15 m (Schrödl 1996a).

##### Type material.

Not available.

##### Distribution.

Abundant in the Magellanic Province, in the Southeast Pacific (Aldea et al. 2011; Velasco‐Charpentier et al. 2021) and with records in the South Atlantic (Eliot 1907; Odhner 1926, 1944), Pacific Ocean (Marcus 1959; Meyers-Muñoz et al. 2009; Schrödl 1996a, 2003), and Indian Ocean (Odhner 1944).

##### Remarks.

It was included in the list of mollusks of Peru by Paredes et al. (1999) as *Flabellinafalklandica* (Eliot 1907); this information was repeated by Ramírez et al. (2003).

According to Uribe et al. (2013), its presence in Peru requires confirmation since the inclusion by Paredes et al. (1999) was based on unfounded records.

#### ﻿ Coryphellina marcusorum

Taxon classificationAnimaliaNudibranchiaPolyceridae

(Gosliner & Kuzirian, 1990)

A2688BA5-FECE-52A3-9425-3B24198D6A1B

##### Habitat.

Benthic.

##### Depth.

3–22 m (Welch 2010).

##### Type material.

CASIZ 066151—San Diego Reef (25°12'N, 110°42'W), Gulf of California, Mexico.

##### Distribution.

From Brazil to Gulf of California (Mexico) (Gosliner and Kuzirian 1990; Fischer et al. 2007).

##### Sampling/reporting sites.

In Peru, this species was recently photographed in Los Organos (Piura, 04°10′S) on 11 March 2022 (Torrejón 2023).

##### Remarks.

Originally named *Flabellinamarcusorum* Gosliner & Kuzirian, 1990. Its presence in Peru needs be confirmed by future surveys.

## ﻿Discussion

### ﻿Overview

This article presents an updated compilation of nudibranchs found in Peru, derived from an extensive literature review. The revised and updated scientific names are presented, while species not verified or erroneously listed in previous articles, referred to here as ’potentially occurring’, are separated from those confirmed.

Despite the presence of two distinct coastal marine biogeographic provinces and a transitional zone between them (Schrödl and Hooker 2014; Ibanez-Erquiaga et al. 2018; Barahona et al. 2019), the species richness of nudibranchs remains relatively low (*n* = 31) compared to other marine regions, such as the Gulf of Mexico, the Caribbean, the South Atlantic, the Indian Ocean, and the Mediterranean Sea (Behrens 2004; Chavanich et al. 2013). In neighboring South American countries, such as Colombia (*n* > 40) (Ardila et al. 2007; Londoño-Cruz 202 1), Chile (*n* > 50) (Schrödl 1996a, 1999b, 2003; Fischer and Cervera 2005b; Schrödl et al. 2005; Schrödl and Grau 2006; Aldea et al. 2011), and Brazil (*n* > 80) (Marcus and Marcus 1969; Padula et al. 2011; Pereira et al. 2012; Padula 2015), a greater diversity of nudibranch species is found (Fig. 3A, B).

It is worth noting that there are areas along the Peruvian coast that remain unexplored. In the Tropical Eastern Pacific, only a limited number of locations have been sampled, including Pocitas, Punta Sal, Mancora, and Cancas. Within the transition zone, Sechura Bay and Foca Island are the common reporting sites, while within the Warm Temperate Southeastern Pacific, Santa, Casma, Huarmey (Ancash), Ancon, Callao, San Lorenzo Island, Pucusana (Lima), Pisco, Independencia Bay, San Juan de Marcona (Ica), Matarani, and Isla Blanca (Arequipa) are frequently mentioned. Factors such as limited exploration efforts, challenging diving conditions, a scarcity of nudibranch taxonomists, and a general lack of interest in this group in Peru should be highlighted. In addition, it is highly likely that several species remain unreported and undescribed, particularly in deeper waters. Therefore, the confirmed number of nudibranch species in Peruvian waters (*n* = 31) is presumed to represent only a fraction of the actual diversity present.

### ﻿Potential influence of the Humboldt Current and El Niño

Several confirmed species exhibit a biogeographical affinity for the Warm Temperate Southeastern Pacific (*n* = 23) (Fig. 2). The Humboldt Current plays a significant role as an oceanographic factor, facilitating the influx of various species from the Chilean sea into Peruvian waters, with 19 of them occurring in both. For instance, *Phidianalottini*, found in Callao and Puerto Malabrigo, may extend into tropical waters due to the influence of the Humboldt Current (Uribe et al. 2013). The occurrence of *Polycerapriva* in Peru came as a surprise to researchers, considering that this species is typically Magellanic and endemic to the Patagonian fjords (Schrödl 1996a, 1999b, 2003, 2009). Therefore, its presence in Peru is attributed to the influence of the Humboldt Current (Schrödl 1996a, 1999b, 2003, 2009; Schrödl and Hooker 2014). Species such as *Corambelucea*, *Coryphellinacerverai*, *Janolusrebeccae*, and *Tritonia* sp., collected in the Warm Temperate Southeastern Pacific province, have also been reported in Sechura Bay and Foca Island, situated in the biogeographical transition zone. This suggests their adaptation to slightly warmer waters (Fig. 4).

El Niño events can induce shifts in the distribution ranges of sea slugs (Goddard et al. 2018) and even result in species turnover (Valqui et al. 2021). During strong El Niño events, it is noteworthy that mollusk species endemic to tropical areas have been observed in Peruvian waters due to the displacement of warm-water masses (Velez and Zeballos 1985; Paredes et al. 1998; Ramírez et al. 2003). Species such as *Arminacalifornica*, *Felimidabaumanni*, *Felimareagassizii*, *Tyrinnaevelinae*, and *Bajaeolisbertschi*, which are abundant in the Tropical Eastern Pacific province, Warm Temperate Northeast Pacific, or Cold Temperate Northeast Pacific, have only been recorded in Tumbes or Piura (the northernmost coastal areas of Peru) (Table 4). This suggests that their presence may be temporary, resulting from the displacement of warm-water masses, or they may have permanently adapted to similar conditions following multiple El Niño events (Ashton et al. 2008).

The distribution patterns of cosmopolitan and circumglobal species can be attributed to various biological factors, including their remarkable dispersal capabilities. Take, for instance, *Glaucusatlanticus*, which possesses intriguing adaptations for dispersal such as larval gas bubbles and specialized anatomy enabling it to exploit water surface tension (Thompson and McFarlane 1967; Valdés and Campillo 2004; Churchill et al. 2014a). Additionally, abiotic factors like ocean currents (Miller 1993) and indirect human influences such as buoy rafting (Astudillo et al. 2009), ballast water, or shipping activities can facilitate the dispersal of these species, expanding their geographic ranges (Borg et al. 2009).

### ﻿Potentially occurring species

This group of species poses a challenge as they have been consistently listed and referenced in several previous articles (indicated by asterisks in Table 3) despite lack of substantiated records. One notable example is *Cadlinasparsa*. Initially mentioned as a probable species by Alamo and Valdivieso (1997), it persisted in the subsequent publications of Paredes et al. (1999), Ramírez et al. (2003), and Nakamura (2006) without acknowledging its speculative status. In the work of Uribe et al. (2013), the authors discussed the predicted occurrence of *Cadlinasparsa* in Peruvian waters based on extrapolation, assuming a continuous distribution, due to its presence in Baja California and Chile.

Paredes et al. (1999), based on personal communication with Sandra Millen, included *Aeolidiacampbellii* (referred to as *Aeolidiaserotina*) and *Itaxiafalklandica* (referred to as *Flabellinafalklandica*), which persisted in the subsequent lists of Nakamura (2006) and Ramírez et al. (2003), respectively. However, no additional published reports have surfaced to substantiate their presence. Ramírez et al. (2003) also included the species *Phylliroelichtensteinii* without providing any justification for its inclusion. On the other hand, Nakamura (2006) mistakenly listed *Gargamellaimmaculata* and *Thecaceradarwini* as present in Peruvian waters, referencing Schrödl (1999b). Nevertheless, Schrödl (1999b) only listed these species in a table as occurring in the ‘zoogeographic Peruvian province’, indicating their presence in the Chilean waters corresponding to this biogeographical province, but not in Peruvian waters.

A solitary specimen of *Coryphellinamarcusorum* was recently documented through photography on the northern coast of Peru (Los Organos, Piura) (Torrejón 2023). Given the species’ tropical distribution and a previous record near countries such as Ecuador (GBIF 2022), its occurrence in Peru is plausible. However, these two records do not provide evidence of an established population in this region, suggesting the possibility that it may have arrived independently through shipping or been displaced by ocean currents. Further collections and taxonomic verifications are necessary to confirm its presence definitively.

Potentially occurring species should not be included in the official list of Peruvian nudibranch species. However, considering their disjointed or patchy geographic distributions or unique observations, they may be reported in Peruvian waters in forthcoming papers. *Rostangapulchra*, for instance, was a long-standing predicted species for Peruvian waters until its initial sighting in San Juan de Marcona (Ica) by Schrödl and Hooker (2014). As a cosmopolitan species, the presence of *Phylliroelichtensteinii* in Peru is plausible. Fischer and Cervera (2005b) have deemed the presence of *Thecaceradarwini* in Peruvian waters highly probable. The term “potentially occurring” signifies a provisional status, indicating that these species have not yet been officially confirmed.

### ﻿Identification uncertainties

The genus *Polycera* displays remarkable color variability, seemingly correlated with its geographic range (Behrens and Hermosillo 2005). A genetic investigation identified clades with overlapping distributions in the Northeastern Pacific, strongly suggesting the existence of a species complex (Santander and Valdés 2013). One year later, a morphology-based study by Pola et al. (2014) revealed that specimens previously collected by Camacho-García et al. (2005) in Costa Rica, Panama, and Mexico actually belong to a new species, *Polyceraanae*. Considering that *P.alabe* was solely “observed” by Millen in Tumbes, it is possible that it represents another *Polycera* species, such as *P.anae*, given its inclusion in a species complex (Santander and Valdés 2013).

The report of *Glaucus* sp., documented by Quesquen (2017), is based on a single poorly preserved specimen sampled in 1995, warranting verification through molecular methods. Currently, within the genus *Glaucus*, only the species *G.atlanticus* has been confirmed, as many previous records turned out to be synonyms (e.g., *G.distichoicus*) or were later reassigned to the genus *Glaucilla*. Similar circumstances apply to the reports of *Tritonia* sp. and *Cuthona* sp., as their species-level recognition is still pending.

### ﻿Concluding remarks

This research contributes to the dissemination and diffusion of this understudied group of organisms in Peru. It is imperative to intensify monitoring efforts to verify the presence of doubtful species, evaluate anthropogenic impacts, and El Niño-driven displacements. Furthermore, considering the intricate nature of external morphological identification, frequent variability in coloration, and the probable existence of cryptic species, it is possible that a considerable number of species remain undiscovered. Consequently, there is an urgent need for comprehensive research involving detailed internal anatomy and the application of molecular tools, such as DNA barcoding and phylogenetic analyses. These methodologies will play a vital role in shedding light on the taxonomy and evolutionary relationships within this group.

## Supplementary Material

XML Treatment for Spurilla braziliana

XML Treatment for Phidiana lottini

XML Treatment for Bajaeolis bertschi

XML Treatment for Glaucus atlanticus

XML Treatment for Armina californica

XML Treatment for Dendronotus cf. venustus

XML Treatment for Doto uva

XML Treatment for Hancockia schoeferti

XML Treatment for Cephalopyge trematoides

XML Treatment for Phylliroe bucephala

XML Treatment for Cuthona

XML Treatment for Fiona pinnata

XML Treatment for Kynaria cynara

XML Treatment for Coryphellina cerverai

XML Treatment for Janolus rebeccae

XML Treatment for Tritonia

XML Treatment for Tyrinna delicata

XML Treatment for Tyrinna evelinae

XML Treatment for Felimare agassizii

XML Treatment for Felimare sechurana

XML Treatment for Felimida baumanni

XML Treatment for Baptodoris peruviana

XML Treatment for Diaulula variolata

XML Treatment for Diaulula punctuolata

XML Treatment for Rostanga pulchra

XML Treatment for Doris fontainii

XML Treatment for Corambe lucea

XML Treatment for Corambe mancorensis

XML Treatment for Okenia luna

XML Treatment for Doriopsilla janaina

XML Treatment for Polycera priva

XML Treatment for Thecacera darwini

XML Treatment for Glaucus

XML Treatment for Aeolidia campbellii

XML Treatment for Gargamella immaculata

XML Treatment for Cadlina sparsa

XML Treatment for Polycera cf. alabe

XML Treatment for Phylliroe lichtensteinii

XML Treatment for Itaxia falklandica

XML Treatment for Coryphellina marcusorum

## ﻿Additional information

### Author ORCIDs

Alessandra Grández https://orcid.org/0000-0002-0142-9357

André Ampuero https://orcid.org/0000-0001-6929-5423

Sergio P. Barahona https://orcid.org/0000-0002-0136-7205

### Data availability

All of the data that support the findings of this study are available in the main text or Supplementary Information.
